# Mechanical Regimes in Gelatin and Gellan Gum Bigels: Structure–Function Relationships and Dual Delivery of Carob Fruit Extracts

**DOI:** 10.3390/gels12070602

**Published:** 2026-07-07

**Authors:** Alicia Gutiérrez, Susana Cofrades, Arancha Saiz, María Dolores Álvarez

**Affiliations:** Institute of Food Science, Technology and Nutrition (ICTAN-CSIC), C/José Antonio Novais 6, 28040 Madrid, Spain; a.gutierrez@ictan.csic.es (A.G.); a.saiz@ictan.csic.es (A.S.)

**Keywords:** bigels, gelatin, gellan gum, rheology, mechanical properties, structure–function relationships, dual delivery, carob fruit extracts

## Abstract

Bigels (BGs) were formulated using gelatin (GA) or gellan gum (GG) hydrogels (HGs) combined with beeswax-structured oleogels (OGs). Carob fruit extracts—an inositol-rich fraction (I-CFE) and a polyphenol-rich fraction (P-CFE)—were incorporated into the HG and OG phases, respectively, to enable dual delivery. The effects of composition on rheological, textural, thermal, color, and stability properties were evaluated at HG/OG ratios of 70/30, 60/40, and 50/50. GG-based BGs formed rigid, coherent, and crystal-reinforced networks, exhibiting the highest oscillatory stiffness and complex viscosity. GA-based BGs developed softer, more deformable, and viscous structures, with mechanical behavior strongly governed by damping and water content. Increasing OG content reinforced GG BGs through beeswax–crystal integration, whereas in GA it increased oscillatory stiffness but weakened the cohesive, viscous, and recoverable characteristics of the protein network. Categorical principal component analysis (CATPCA) revealed two mechanical domains: a GA-associated regime dominated by viscosity, penetration resistance, and loss factor (tan *δ*), and a GG-associated regime governed by elastic stiffness. Correlations confirmed tan *δ*_max_ as a marker of structural fragility in GA, while stiffness parameters dominated GG behavior. Melting points remained within 53–54 °C, and all BGs showed excellent physical stability. Overall, GA and GG provide complementary design spaces, offering a mechanistic basis for the rational design of BGs with controlled structural and functional properties.

## 1. Introduction

Bigels (BGs) are biphasic soft materials composed of a hydrophilic hydrogel (HG) and a lipophilic oleogel (OG), each forming its own three-dimensional network [1]. Their dual-phase architecture enables the simultaneous encapsulation of hydrophilic and lipophilic compounds, offering advantages over emulsions and single-phase gels, such as improved stability, tunable mechanical properties, and surfactant-free structuring [1,2]. Initially developed for pharmaceutical and cosmetic applications, BGs are now gaining relevance in food science as fat replacers and delivery systems aligned with healthier and more sustainable formulations [3,4].

BG structure depends strongly on phase composition, the hydrogel-to-oleogel ratio (HG/OG), and processing conditions. These factors determine whether BGs adopt O/W, W/O, or bicontinuous architectures [5], and these architectures govern their mechanical strength, viscoelasticity, and thermal stability. HG-rich systems typically form O/W structures [6,7], whereas higher OG contents may induce phase inversion or interpenetrating networks [8,9].

Gelatin (GA) is widely used in food-grade BGs due to its thermoreversible gelation, elasticity, and water-binding capacity, often producing uniform droplet distributions [6]. GA-based BGs have been explored in 3D printing and hybrid matrices with ethyl cellulose or gellan gum (GG), where the HG/OG ratio and emulsifier selection modulate mechanical and physicochemical properties [4,10,11,12]. Additional biopolymers, such as κ-carrageenan, CMC, or konjac glucomannan, can further modify HG networks through electrostatic or viscosity-driven interactions [13,14,15].

OGs structured with natural waxes (e.g., beeswax (BW), rice bran wax, and candelilla wax) provide crystalline networks that reinforce the lipid phase and contribute to thermal transitions between 40–80 °C [16,17]. BW-based OGs are particularly compatible with GA and widely used in food BGs [18,19,20,21]. Increasing OG content often enhances rigidity and viscoelastic moduli [7,22], whereas HG-rich systems tend to be softer and more deformable [6,9]. However, structural transitions in GA–wax BGs at intermediate ratios remain insufficiently understood. In this context, GA and GG were selected as HG-forming agents because they represent two widely used and mechanistically distinct food-grade gelling systems (protein-based and polysaccharide-based), while BW was chosen as the oleogelator due to its strong ability to form crystalline lipid networks at relatively low concentrations and its broad use in food OGs. This combination enables the design of hybrid BG matrices that are technologically relevant and compatible with the incorporation of carob fruit extracts.

Beyond their technological interest, BGs are promising carriers for bioactive compounds, enabling sequential release and improved stability of hydrophilic and lipophilic molecules, such as carotenoids, phenolics, and saponins [12,23,24,25]. Their biphasic nature enhances protection during digestion and modulates release kinetics [15,25].

Carob fruit extracts (CFEs) are emerging functional ingredients relevant to diabesity management [9]. The carob pod contains an inositol-rich fraction (I-CFE), dominated by pinitol, and a polyphenol-rich fraction (P-CFE), rich in proanthocyanidins with antioxidant and gut-protective effects. Antagonistic absorption pathways and phenolic instability [26,27] limit their incorporation into foods. Embedding I-CFE in the HG and P-CFE in the OG of BGs enables sequential release [9], reduces unwanted interactions, and improves oxidative and sensory stability. Carob fruit (*Ceratonia siliqua* L.) contains several functional ingredients of technological and nutritional relevance. The pod is naturally rich in inositols, mainly D-pinitol and myo-inositol, which can reach concentrations of 145–155 g/kg in concentrated extracts, as well as polyphenols (condensed tannins and gallic acid derivatives) and dietary fiber composed of cellulose, hemicellulose, and pectins. The seed (kernel) contains high levels of galactomannans (locust bean gum), together with proteins and structural polysaccharides. These polymeric species contribute to the viscosity, water-binding capacity, and antioxidant potential of carob-derived ingredients.

Given these gaps, this work developed and characterized food-grade BGs formulated with GA- and GG-based HGs and BW-based OGs at HG/OG ratios of 70/30, 60/40, and 50/50. This work builds upon our previous study [9] on alginate-based carob extract BGs by introducing a comparative analysis of two mechanistically distinct HG-forming agents (GA and GG) and by examining how their contrasting gelation pathways give rise to different mechanical regimes, structural organization, and dual-phase delivery behavior across intermediate HG/OG ratios. GA and GG were selected because they represent two widely used and mechanistically distinct food-grade gelling systems, which were expected to generate contrasting structural responses. I-CFE was incorporated into the HG and P-CFE into the OG to enable controlled sequential release during in vitro digestion. These ratios were selected to cover the transition from HG-dominated structures to more balanced biphasic networks. We hypothesized that GA- and GG-based BGs would exhibit distinct mechanical regimes driven by their different gelation mechanisms, and that the HG/OG ratio would modulate these regimes and the dual-phase delivery performance of carob fruit extracts.

## 2. Results and Discussion

### 2.1. SAOS Measurements of BGs

#### 2.1.1. Stress Sweep Tests

**Figure 1:**
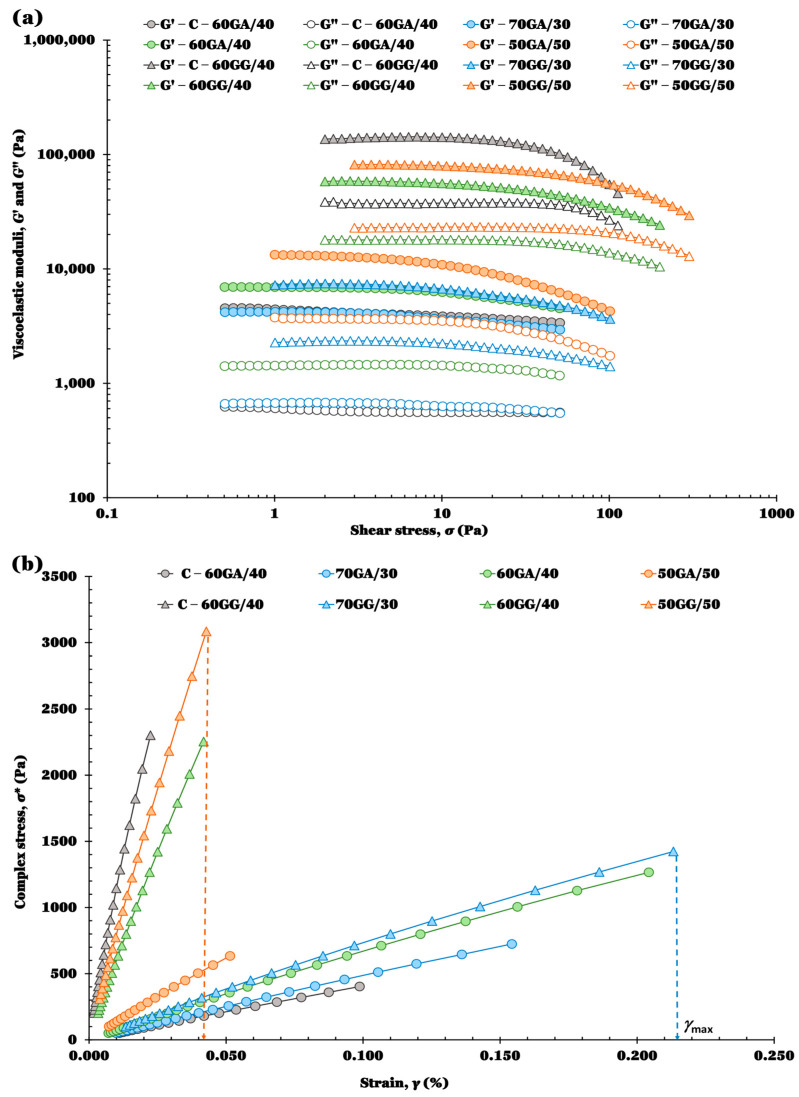
(**a**) Stress-sweep curves of bigels (BGs) formulated with gelatin (GA) and gellan gum (GG) at different HG/OG ratios (50/50, 60/40, 70/30). Storage modulus (*G*′, filled symbols) and loss modulus (*G*″, open symbols) are plotted as a function of applied shear stress (*σ*) to identify the linear viscoelastic region (LVR) of each formulation. (**b**) Complex shear stress (σ*) as a function of strain (*γ*) of BGs formulated with GA and GG at 50/50, 60/40, and 70/30 ratios.

The viscoelastic properties of the BGs were first assessed through stress sweep tests (Figure 1) to determine the linear viscoelastic region (LVR) limits. All formulations showed a predominant solid-like behavior within this region (*G*′ > *G*″, tan *δ* < 1), confirming their classification as weak gels [14]. BGs containing GG exhibited substantially higher *G*′ and *G*″ values than those formulated with GA, reflecting the formation of a more rigid and cohesive polysaccharide-based network [4,6,12]. This difference reflects the combined influence of polymer type and the concentrations typically required for effective gelation of GA and GG systems. This response is characteristic of gellan systems, where the ionic double-helix aggregation, a structural mechanism widely reported for GG systems [12], and crystalline domains from BW or other oleogelators contribute to stiff, brittle structures [8,21], as supported by the BW crystals observed under polarized light microscopy in this study.

For both hydrogelifiers, G′ and G″ values decreased as the HG fraction increased (70/30) compared to the 60/40 and 50/50 ratios (Figure 1a). These differences arise from the combined effects of hydrogelifier type and HG/OG ratio, which modulate the internal architecture of the BGs. Similar ratio-dependent transitions have been reported in GG–BW systems, where increasing the OG fraction reinforces the network through progressive crystal integration [4,28]. Overall, viscoelastic moduli increased as the water and hydrogelifier contents decreased in the HG phase, while the OPO, BW, and lecithin contents increased in the OG phase, as described in Section 4.2, consistent with the contribution of crystalline OG networks to BG rigidity [16,17,18]. In general, GG-based BGs behaved as stronger but more brittle systems, whereas GA-based BGs were softer and more deformable, reflecting the contrast between rigid ionic polysaccharide networks and more dissipative protein-based gels [13,29].

The I-CFE and P-CFE contents were fixed at 30% in the HG and 1% in the OG, respectively, as detailed in Section 4.2. Thus, for a given HG/OG ratio, BGs formulated with GA or GG shared the same OG composition, and the rheological differences arose solely from the water content and the type and concentration of polymer in the HG. For instance, although the 50GA/50 BG contained less water (16.43%) and more polymer (3.57% GA) than the 50GG/50 BG (19.50% water, 0.50% GG), the latter exhibited much higher viscoelastic moduli, highlighting the superior gel-forming capacity and stiffness of GG-based HGs [13,15,29]. Similar behavior has been widely reported in gellan systems, where even low GG concentrations generate dense, highly elastic networks that surpass protein-based HGs in rigidity [4,25].

At the 60/40 ratio (Figure 1), extract-free BGs (control formulations) showed contrasting behaviors depending on the hydrogelifier. In GA systems, the control 60GA/40 exhibited lower viscoelastic moduli than the extract-loaded BG, suggesting that I-CFE and P-CFE reinforced the network, likely through additional polymer–polymer or polymer–solute interactions. This strengthening effect aligns with reports describing phenolic-induced enhancement of protein gels [13,29]. Conversely, in GG systems, the control 60GG/40 displayed higher moduli than the extract-loaded BG and even exceeded the 50GG/50 formulation, indicating that the extracts did not reinforce the GG network. Instead, they may have interfered with OG crystallinity or with GG ionic crosslinking, yielding slightly weaker structures—an effect previously observed when solutes disrupt crystal packing or ionic associations in GG-based BGs [21,25].

Table 1 summarizes the critical rheological parameters defining the LVR of the BGs. Control formulations at the 60/40 ratio (without extracts) are included only as reference values and were not considered in the statistical analysis. They provide baseline mechanical profiles for GA- and GG-based systems. The maximum stress (*σ*_max_) and strain (*γ*_max_) indicate the mechanical limits before structural failure, while tan *δ*_max_ reflects the elastic–viscous balance at the end of the LVR.

In GA systems, *σ*_max_ values increased significantly from 50_GA_/50 to 60_GA_/40 (*p* < 0.05), indicating that a moderate rise in the HG fraction strengthened the network, but decreased again at 70_GA_/30 (*p* < 0.05), where the higher aqueous phase weakened the structure. *γ*_max_ values followed the same significant pattern (*p* < 0.05), with 60_GA_/40 showing the highest deformation tolerance. Conversely, tan *δ*_max_ values decreased significantly with increasing HG content (*p* < 0.05), reflecting a more elastic response. In GG systems, *σ*_max_ values increased significantly from 70_GG_/30 to 50_GG_/50 (*p* < 0.05), confirming that higher OG content consistently reinforced the network. *γ*_max_ values remained low for 50_GG_/50 and 60_GG_/40 (no significant difference between them), indicating brittle behavior, whereas 70_GG_/30 showed a higher *γ*_max_ value due to its greater water content and lower OG crystallinity. tan *δ*_max_ values did not differ significantly among GG formulations, suggesting that the HG/OG ratio had little influence on the elastic–viscous balance in these systems.

For each HG/OG ratio, GG-based BGs showed significantly higher *σ*_max_ values than GA-based ones (*p* < 0.05), confirming the greater rigidity of GG networks. By contrast, *γ*_max_ values were consistently higher in GA systems (*p* < 0.05)—especially at 60/40—reflecting their greater deformability. Regarding tan *δ*_max_, values were significantly higher in GG formulations at 70/30 and 60/40 (*p* < 0.05), whereas no significant difference was observed at 50/50. Overall, these trends reinforce the contrast between the softer, more viscous GA networks and the stiffer, more brittle GG structures.

To further characterize the transition from linear to nonlinear behavior, the evolution of complex shear stress (σ*) with strain (*γ*) was examined (Figure 1b). The initial linear region of each *σ*–*γ* curve was fitted with Equation (1) to obtain parameters *a* and *b*, which describe the resistance at very small deformations. From Figure 1b, 70_GG_/30, 60_GA_/40, and 70_GA_/30 showed the greatest conformational flexibility (>0.150%), whereas 50_GG_/50 and 60_GG_/40 behaved as the most rigid systems.

GG-based BGs exhibited markedly higher *a* and *b* values than GA-based ones (*p* < 0.05), confirming their stronger resistance to deformation (Table 1). Within GG systems, both parameters increased with OG content, reflecting progressive network reinforcement. By contrast, the energy parameter *E* followed a different pattern: its highest value occurred in 70_GG_/30 due to its larger *γ*_max_ value, indicating greater toughness and energy absorption before failure. A similar trend was observed in GA BGs. Although *a* values increased with OG proportion, *E* values did not; instead, 60_GA_/40 showed the highest *E* value, combining moderate stiffness with high deformation tolerance. This behavior aligns with reports showing that intermediate HG/OG ratios maximize toughness by balancing rigidity and flexibility. Conversely, 50_GA_/50 exhibited the highest *a* value but the lowest *E* value, indicating a rigid but brittle structure, while 70_GA_/30 was softer and more flexible, yielding intermediate *E* values.

Overall, these results indicate that 70_GG_/30, 60_GA_/40, and 70_GA_/30 do not behave as purely stiffness-driven systems but instead achieve a balance between rigidity and deformability that enhances their capacity to absorb mechanical energy.

#### 2.1.2. Frequency Sweep Tests

**Figure 2:**
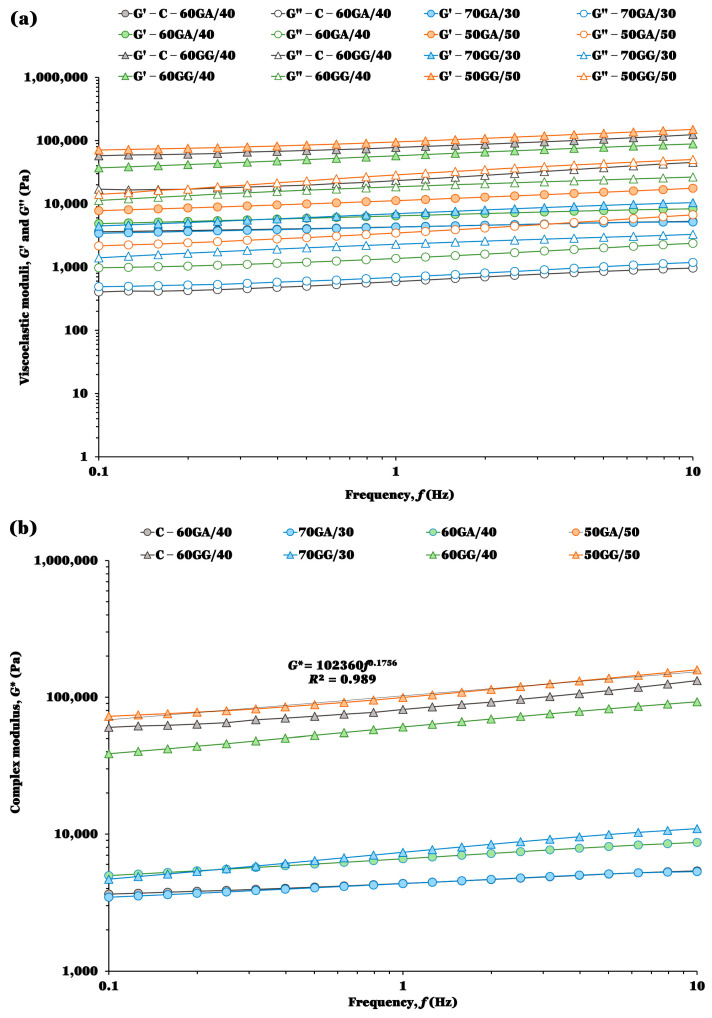
(**a**) Mechanical spectra of bigels (BGs) formulated with gelatin (GA) and gellan gum (GG) at different HG/OG ratios (50/50, 60/40, 70/30). Storage modulus (*G*′, filled symbols) and loss modulus (*G*″, open symbols) are plotted as a function of applied frequency (*f*). (**b**) Complex modulus (G*) vs. frequency for fits to weak-gel model of BGs formulated with GA and GG at 50/50, 60/40, and 70/30 ratios.

Frequency sweep measurements provided further insight into the viscoelastic behavior of the BGs (Figure 2). In all formulations, *G*′ values exceeded *G*″ values across the tested range, confirming their gel-like nature. GG-based BGs showed only minor changes in the *G*′/*G*″ ratio, reflecting a frequency-insensitive, strongly elastic network typical of gellan systems reinforced by double-helix junction zones and crystalline OG domains [4,12]. By contrast, GA-based BGs exhibited a progressive widening of the *G*′–*G*″ gap as the HG fraction increased, indicating a shift toward more elastic behavior, consistent with protein-based composite gels [13].

Table 2 summarizes the mechanical spectra at 1 Hz and the weak-gel model parameters. As in the stress sweeps, the 60/40 controls (without extracts) are included only as reference values. Their behavior paralleled previous observations: the control 60_GA_/40 was softer than its extract-loaded counterpart, whereas the control 60_GG_/40 was markedly stronger—exceeding even 50_GG_/50 in several parameters. These opposite effects indicate that the extracts reinforce GA networks but slightly weaken GG ones, consistent with reports showing that phenolic compounds strengthen protein-based gels but may interfere with crystallinity or ionic crosslinking in GG systems [21].

Increasing the OG fraction reinforced both systems, but with distinct patterns. In GA BGs, *G*′, *G*″ and η* values increased significantly with OG content (*p* < 0.05), while tan *δ* values also rose significantly (*p* < 0.05), indicating that stiffness gains were accompanied by higher viscous dissipation. This trend mirrors the stress-sweep results, where 50_GA_/50 behaved as a stiff but less elastic formulation, whereas 60_GA_/40 maintained a more balanced profile [4]. In GG BGs, *G*′ and *G*″ values increased significantly from 70_GG_/30 to 50_GG_/50 (*p* < 0.05), but tan *δ* values remained nearly constant (no significant differences), confirming the inherently stable and elastic nature of GG networks [30]. η* values followed the same significant trend (*p* < 0.05), particularly in 50_GG_/50, which formed the most cohesive structure.

The mechanical spectra of all BGs were accurately fitted to the weak-gel model (Figure 2b). The linear log G*–log *f* dependence confirmed that their viscoelastic response was governed by interacting flow units, a characteristic of partially interpenetrated biphasic gels [31,32]. High *R*^2^ values (≥0.984) validated the applicability of the model, in agreement with previous reports on GG–BW bigels [4].

In GA-based BGs (Table 2), the interaction strength *A* increased significantly with OG content (*p* < 0.05), whereas z values decreased significantly (*p* < 0.05). Since the frequency exponent is 1/*z*, lower *z* values indicate stronger frequency dependence and mechanically stiffer but less extended networks. Thus, the significant decrease in *z* value from 70_GA_/30 to 50_GA_/50 reflects a transition toward fewer cooperative flow units and a more compact, rigid structure, consistent with GA composite gels where reduced aqueous structuring enhances stiffness [13]. In GG-based BGs, *A* values also increased significantly with OG proportion (*p* < 0.05), but *z* values remained low and nearly constant (no significant differences), confirming that GG networks maintain a similar degree of structural extension regardless of composition. This invariance is typical of GG systems, where the ionic double-helix network remains structurally dominant even when the OG phase increases [6,21].

At equivalent HG/OG ratios, GG-based BGs exhibited significantly higher *G*′, *G*″, η*, and *A* values than GA-based ones (*p* < 0.05), confirming that GG forms a more cohesive and mechanically robust network even at lower polymer concentration. By contrast, GA formulations—particularly 70_GA_/30 and 60_GA_/40—showed significantly higher 1/*z* values (*p* < 0.05), reflecting more deformable and structurally dynamic networks with a larger number of interacting flow units. These differences highlight the dominant role of the hydrogelator in defining the mechanical identity of the BGs, consistent with comparative studies of protein- and polysaccharide-based hydrogels [13,29].

Taken together, the combined analysis of frequency sweeps and weak-gel modeling reinforces the structural trends identified in the LVR. GG-based BGs form strong, densely connected networks with low frequency dependence, whereas GA-based BGs exhibit weaker but more flexible architectures. Notably, formulations such as 70_GG_/30, 60GA/40, and 70_GA_/30 also stood out in the stress sweep tests due to their higher energy storage capacity (*E*). This convergence indicates that these BGs achieve a distinct balance between rigidity and deformability, combining elastic dominance with enhanced structural adaptability. Such balanced architectures have been associated with improved performance in sequential release of hydrophilic and lipophilic compounds [15,25].

### 2.2. Flow and Thixotropic Behavior of BGs

**Figure 3:**
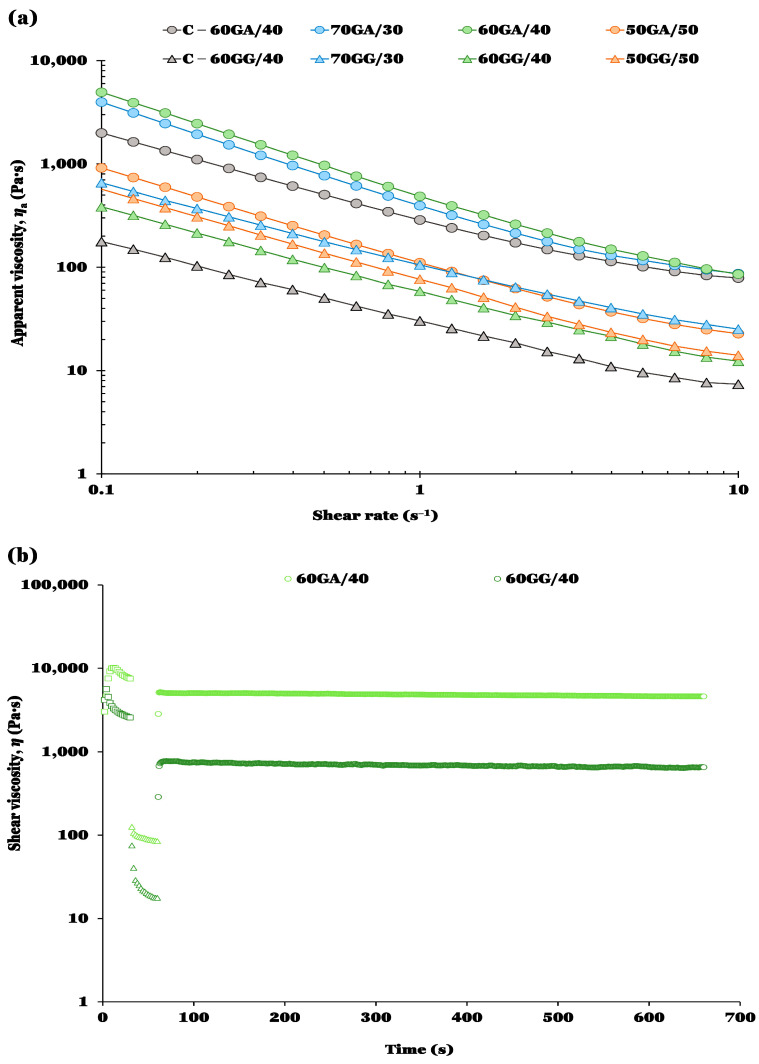
(**a**) Apparent viscosity of bigels (BGs) formulated with gelatin (GA) and gellan gum (GG) at different HG/OG ratios (50/50, 60/40, 70/30). (**b**) Viscosity as a function of time from a three-step shear test of BGs formulated with GA and GG at 50/50, 60/40, and 70/30 ratios. Squares and triangles correspond to the first and second shear steps (0.1 s^−1^ for 30 s and 10 s^−1^ for 30 s, respectively), while circles represent the third step (0.1 s^−1^ for 600 s) during viscosity recovery.

The flow behavior of the BGs was strongly influenced by both the hydrogelator and the HG/OG ratio. All formulations exhibited shear-thinning behavior (Figure 3a), consistent with the progressive disruption and alignment of biphasic networks under flow [16]. Across the entire shear-rate range, GA-based BGs showed substantially higher apparent viscosities than GG-based ones, reflecting the denser and more hydrated nature of GA networks [29].

Table 3 summarizes the steady-shear parameters. The 60/40 controls (without extracts) displayed lower viscosities than their extract-loaded counterparts, particularly in GG, indicating that the extracts enhanced flow resistance in both systems, with a stronger structuring effect in GG BGs [21]. Within the GA group, viscosity and the consistency index K followed the order 60_GA_/40 > 70_GA_/30 ≫ 50_GA_/50, confirming that the intermediate ratio produced the most viscous system. *η_0_*_.1_ and *K* values were significantly higher in 60_GA_/40 and 70_GA_/30 than in 50_GA_/50 (*p* < 0.05). The flow index *n* remained low in all GA formulations, with 60_GA_/40 showing the strongest pseudoplasticity; *n* values differed significantly among GA ratios (*p* < 0.05). The sharp decrease in viscosity and *K* values in 50_GA_/50 reflects the sensitivity of gelatin networks to water availability: reduced aqueous phase limits triple-helix formation and weakens the network [13].

GG-based BGs showed a different pattern. 70_GG_/30 exhibited the highest viscosities, consistent with its larger HG fraction, whereas 60_GG_/40 showed the lowest values due to dilution of the GG network. *η*_0.1_ and *η*_10_ values were significantly higher in 70_GG_/30 than in 60_GG_/40 and 50_GG_/50 (*p* < 0.05), while no significant difference was observed between 60_GG_/40 and 50_GG_/50. The 50_GG_/50 formulation displayed intermediate viscosity but the lowest *n* value; the *n* value was significantly lower in 50_GG_/50 compared to 70_GG_/30 and 60_GG_/40 (*p* < 0.05). These trends align with reports showing that increasing OG can reduce viscosity by diluting the HG network and increasing droplet mobility [33]. Conceptually, these results highlight a key distinction between hydrogelators: GG forms rigid, crystal-reinforced networks that dominate oscillatory behavior, whereas GA networks—more hydrated and deformable—generate higher viscous resistance under steady shear.

Figure 3b illustrates the thixotropic behavior of the BGs using the 60/40 formulations as representative examples. All samples showed a pronounced viscosity drop under high shear followed by rapid recovery once the shear rate returned to 0.1 s^−1^, indicating largely reversible structural breakdown. Recovery values (Table 3) further highlight the influence of formulation variables. In GA BGs, recovery followed the order 60_GA_/40 ≈ 70_GA_/30 ≫ 50_GA_/50; recovery was significantly lower in 50_GA_/50 than in 60_GA_/40 and 70_GA_/30 (*p* < 0.05). In GG BGs, recovery followed the order 70_GG_/30 > 50_GG_/50 > 60_GG_/40; recovery was significantly lower in 60_GG_/40 than in 70_GG_/30 and 50_GG_/50 (*p* < 0.05). The lowest recovery in 60_GG_/40 suggests that this intermediate ratio produces the least resilient structure, likely because neither phase dominates the network sufficiently to sustain integrity after deformation. Overall, GG systems showed more moderate and composition-independent recovery, consistent with their rigid, crystal-reinforced networks, which resist deformation but reorganize slowly [4].

When comparing hydrogelators within each ratio, GA BGs exhibited significantly higher viscosities, higher *K* values and higher recovery percentages than GG BGs (*p* < 0.05), except at 50/50, where GG showed slightly higher recovery (no significant difference). This general superiority of GA in structural rebuildability reflects its ability to form highly hydrated and flexible networks capable of rapid re-association after deformation [13]. The exceptional recovery of 60_GA_/40—the highest among all formulations—highlights the presence of an optimally balanced biphasic structure in which the GA network remains dominant while the OG phase contributes mechanical reinforcement without excessively hindering reorganization.

Collectively, these findings reinforce that network flexibility, hydration level, and HG dominance are key determinants of recoverability in biphasic gel systems, in agreement with recent studies on GG- and BW-based BGs [21,25].

### 2.3. Textural Properties of BGs, HGs, and OGs

Penetration tests were performed on BGs, HGs, and OGs at 5 °C to evaluate their large-deformation mechanical behavior. Hardness (*F*_10_), work of penetration (*W*_10_), breaking force (*F*_B_), and breaking slope (*S*_B_) were obtained, providing complementary information on resistance, cohesiveness, and fracture behavior. In both GA and GG matrices, the 60/40 controls showed lower hardness and work than their extract-loaded counterparts, indicating that the extracts reinforced the mechanical structure of BGs, HGs, and OGs, consistent with the strengthening effect of phenolic compounds in protein- and polysaccharide-based gels [13].

**Figure 4:**
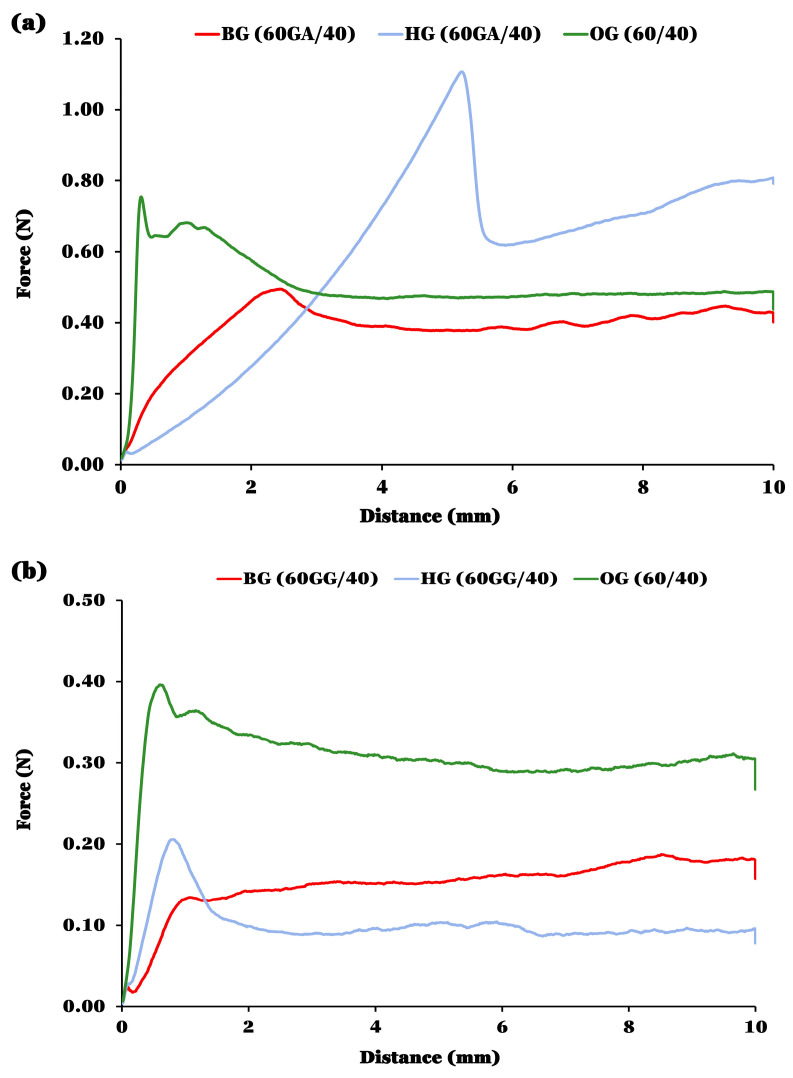
(**a**) Typical force–distance curves obtained from penetration tests for the BGs, HGs, and OGs at the 60/40 ratio using GA as hydrogelator. (**b**) Typical force–distance curves obtained from penetration tests for the BGs, HGs, and OGs at the 60/40 ratio using GG as hydrogelator.

Figure 4 illustrates representative force–distance curves for the 60/40 systems. In GA matrices, the HG showed the highest forces and a smooth, continuous profile, whereas the OG exhibited the steepest initial slope due to BW crystals, and the BG displayed intermediate behavior. In GG matrices, the HG was much softer, the OG again showed the steepest slope, and the BG closely resembled the GG HG. These qualitative differences anticipate the quantitative trends and reflect the higher cohesiveness of GA networks compared with the softer, more brittle GG structures [4].

Table 4 summarizes the textural parameters for all systems. In GA-based BGs, all textural parameters (*F*_10_, *W*_10_, *F*_B_, and *S*_B_) decreased from 70_GA_/30 to 50_GA_/50, with significant differences between 50_GA_/50 and the two higher-HG formulations (*p* < 0.05), reflecting the weakening of the GA network as water and gelatin contents decreased. The OG phase did not compensate for this loss, as BW crystals form a discontinuous dispersed phase within GA matrices [18]. This hydration-dependent weakening is characteristic of GA systems, where reduced water availability limits triple-helix formation and decreases mechanical resistance [13]. By contrast, GG-based BGs showed a clear reinforcement effect with increasing OG fraction: 70_GG_/30 was the weakest, whereas 50_GG_/50 exhibited the highest hardness and stiffness. All textural parameters differed significantly across GG ratios (*p* < 0.05), reflecting the increasing continuity of the BW crystal network and its strong interaction with the GG matrix [9].

At all ratios, GA BGs exhibited significantly higher hardness, work of penetration, and breaking force than GG BGs (*p* < 0.05), reflecting the cohesive and hydrated nature of the GA network (Table 4). GG BGs transitioned from weak and brittle at high HG contents to rigid and crystal-reinforced at high OG contents. At the 50/50 ratio, both systems reached similar hardness values, with no significant difference between hydrogelators at this ratio, but through opposite mechanisms: GA BGs weakened due to HG disruption, whereas GG BGs strengthened due to OG continuity and crystal packing [29].

The behavior of the HGs reflected the intrinsic mechanical differences between GA and GG. GA HGs showed substantially higher *F*_10_, *W*_10_, and *F*_B_ values than GG HGs, with significant differences between hydrogelators at 70/30 and 60/40 (*p* < 0.05). Reducing water content to 50/50 caused a marked decrease in all parameters, significantly lower than the values at 70/30 and 60/40 (*p* < 0.05), consistent with hydration-dependent weakening [14]. GG HGs were initially very soft at high water contents (70/30), but their mechanical resistance increased sharply as the HG fraction decreased; *F*_10_, *W*_10_, and *F*_B_ values were significantly higher in HG_50GG/50_ than in HG_70GG/30_ and HG_60GG/40_ (*p* < 0.05), reflecting compaction of the ionically cross-linked GG network [34].

OGs exhibited the highest stiffness values among all phases, confirming the formation of a dense crystalline network typical of BW-structured lipid systems [18]. Variations in stiffness reflected differences in crystal packing and deformation modes, with significant differences among OG ratios (*p* < 0.05), consistent with the rigid, needle-like BW crystal networks described in previous studies [21].

Across all matrices, the extracts exerted a reinforcing effect, although the underlying mechanisms differed. In GA systems, the extracts increased *S*_B_ significantly (*p* < 0.05) by enhancing network cohesiveness and energy dissipation, whereas in GG systems the increase reflected greater stiffness and fracture resistance due to the more rigid polysaccharide–crystal structure. This dual behavior aligns with reports showing that phenolic compounds strengthen protein networks through additional interactions, while in polysaccharide-based systems they may promote crystal packing or modify interfacial structuring [13].

### 2.4. Multivariate Analysis (CATPCA) of Rheological and Textural Behavior in GA and GG Systems

**Figure 5:**
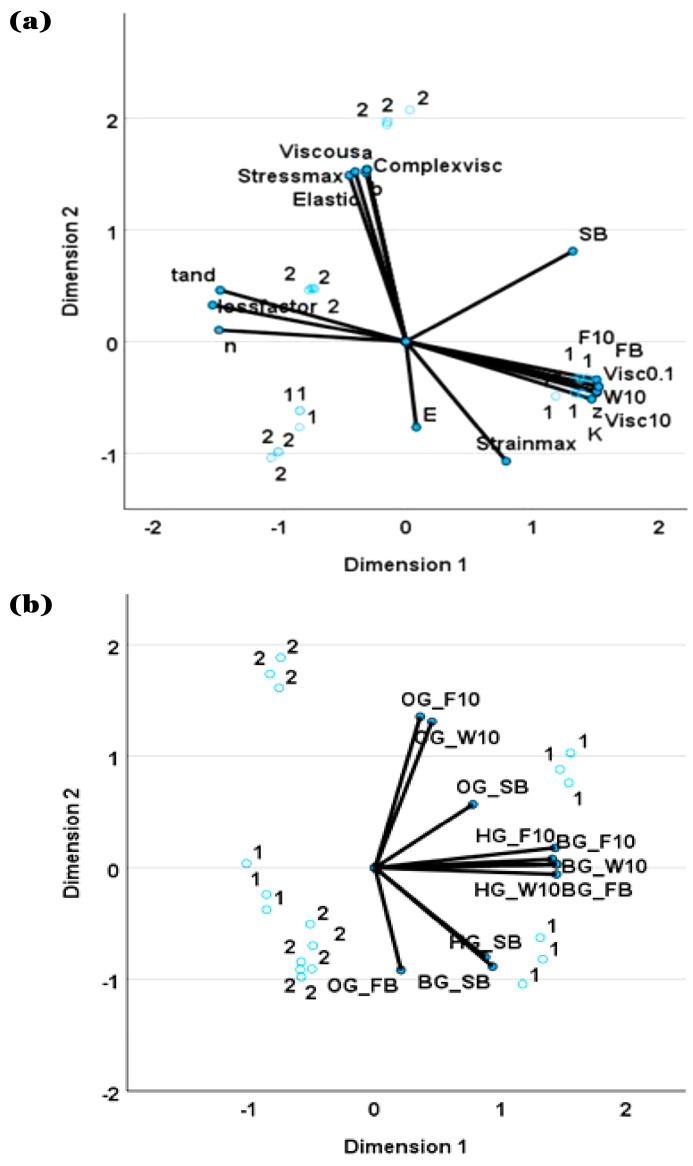
(**a**) Biplot-CATPCA indicating the observed cases (blue dots: BGs formulated with GA (1) and BGs formulated with GG (2)) and component loadings of the rheological and textural properties and parameters analyzed in BGs (black lines). The variable names displayed in the plot correspond to the labels used for each parameter in the CATPCA analysis: Stressmax = *σ*_max_ (critical shear stress), Strainmax = *γ*_max_ (critical shear strain), tand = tan *δ* (loss factor within the LVR), *a* = gel strength, *b* = *σ*_0_ (initial stress), *E* = area under the *σ*_max_–*γ*_max_ line; Elastic = *G*′ (storage modulus), Viscous = *G*″ (loss modulus), loss factor = tan *δ* (loss factor), Complexvisc = η* (complex viscosity), *A*mod = *A* (interaction strength), *z* = network extension; Visc0.1 = *η*_0.1_ and Visc10 = *η*_10_ (apparent viscosities at 0.1 and 10 s^−1^); *K* = consistency index, *n* = flow behavior index; *F*_10_ = force at 10 mm, *W*_10_ = total work up to 10 mm, *F*_B_ = breaking force at the first rupture peak, and *S*_B_ = slope at the first rupture peak. (**b**) Biplot-CATPCA showing the observed cases and the component loadings of the textural parameters evaluated in BGs, HGs, and OGs (*F*_10_, *W*_10_, *F*_B_, and *S*_B_ for each gel type).

Figure 5a shows the CATPCA performed on all rheological and textural parameters of the extract-containing BGs (controls excluded to avoid compositional bias). The analysis revealed a clear separation between GA-based (GA = 1) and GG-based (GG = 2) BGs, with the first two dimensions explaining 92.8% of the variance (66.9% and 26.0%, respectively) and excellent internal consistency (Cronbach’s *α* = 0.996). This strong segregation aligns with previous reports describing the fundamentally different mechanical regimes of GA- and GG-structured bigels [13,29].

Dimension 1 represented a coupled penetration–viscosity–flow–recovery axis, with high positive loadings for penetration forces (*F*_10_, *W*_10_, *F*_B_, and *S*_B_), steady-shear viscosities (*η*_10_ and *η*_0.1_), *K*, recovery, and *z*, and negative loadings for *n* and tan *δ*. GA The 70_GA_/30 and 60_GA_/40 BGs scored positively, reflecting their higher viscosity, penetration resistance, and damping-dominated behavior, whereas 50_GA_/50 scored negatively, consistent with the hydration-dependent weakening of GA networks [14,29].

Dimension 2 captured oscillatory stiffness and elastic coherence, driven by *σ*_max_, parameters *a* and *b*, *G*′, *G*″, η*, and *A*. The 50_GG_/50 and 60_GG_/40 BGs scored positively, indicating more cohesive and rigid elastic architectures, while 70_GG_/30 scored negatively, consistent with its lower viscoelastic moduli. This pattern reflects the strong, frequency-insensitive elastic networks characteristic of GG and BW-based OGs [4,12].

Overall, the CATPCA demonstrates that GA and GG BGs occupy two distinct mechanical regimes: GA systems are dominated by viscous dissipation, localized deformation resistance, and flow-related responses, whereas GG systems are defined by elastic stiffness, structural coherence, and oscillatory resilience. This multivariate separation reinforces the dual mechanical behavior identified in the univariate rheological and textural analyses and is consistent with the contrasting architectures of protein-based vs. polysaccharide-crystal networks [8,25].

Figure 5b shows the CATPCA of textural attributes from BGs, HGs, and OGs. Dimension 1 was associated with penetration forces and work of BGs and HGs, defining a localized deformation axis. Dimension 2 was driven by OG-specific variables, reflecting the crystalline structuring of OGs [18,21]. GA BGs spanned both sides of Dimension 1, with 50_GA_/50 being the softest and 60_GA_/40–70_GA_/30 being the firmest. GG BGs aligned mainly with Dimension 2: 50_GG_/50 and 60_GG_/40 clustered negatively due to higher OG rupture forces, whereas 70_GG_/30 shifted positively, co-locating with OG textural variables, consistent with the increasing contribution of OG crystal packing as the OG fraction rises [6,8].

### 2.5. Correlation Analysis Within GA and GG Mechanical Regimes

Because CATPCA revealed two clearly differentiated mechanical regimes, correlation analyses were performed separately for GA and GG BGs to avoid masking system-specific structure–function relationships. The resulting patterns confirmed that GA and GG exhibit fundamentally distinct mechanical architectures linking oscillatory, steady-shear and penetration responses. GA BGs (Table S1) showed an exceptionally coherent architecture in which critical amplitude parameters (*σ*_max_, γ_max_, *a*, *b*, and tan *δ*_max_) correlated almost perfectly with linear viscoelastic moduli and complex viscosity (*G*′, *G*″, and η*). This indicates that the linear network strongly governs nonlinear deformation. tan *δ*_max_ emerged as a sensitive marker of structural weakening, displaying strong negative correlations with steady-shear viscosity (*η*_10_ and *η*_0.1_), consistency (*K*), recovery, the structural parameter *z*, and penetration resistance (*F*_10_, *F*_B_, *S*_B_, and *W*_10_). Thus, GA BGs with higher damping—particularly 50_GA_/50—exhibited lower viscosity and reduced textural strength. This tight coupling between damping, viscosity and failure is characteristic of protein-based gels, where viscous dissipation drives mechanical fragility [13,29].

GG BGs (Table S2) displayed a rigid, stiffness-dominated architecture. Stress-sweep parameters and linear viscoelastic moduli were nearly collinear (*r* ≈ 0.95–1.00), reflecting a monolithic network governed by elastic rigidity rather than damping. Unlike GA, tan *δ*_max_ showed weak or moderate correlations with viscoelastic, flow and textural variables, indicating that damping plays a minor role in GG. Penetration resistance correlated strongly with oscillatory stiffness (*σ*_max_, *a*, *b*, *G*′, *G*″, and η*) and with steady-shear viscosities and consistency (*η*_10_, *η*_0.1_, and *K*), while the flow index *n* correlated negatively with penetration forces. This behavior is typical of GG-based systems, where ionic double-helix junction zones and crystalline fillers dominate mechanical performance [4,12].

Taken together, these results show that GA and GG represent two distinct mechanical regimes. GA exhibits a flexible but coherent architecture in which damping-related weakening tightly controls flow behavior and mechanical failure. By contrast, GG displays a stiffness-driven regime in which elastic rigidity governs both oscillatory and textural responses. This mechanistic divergence aligns with previous comparisons between protein-based and polysaccharide-based bigels [25,29].

Correlations between BG, HG, and OG penetration parameters (Tables S3 and S4) further support this dichotomy. Because OGs share the same formulation across all BGs, their internal variability is limited, and OG–OG correlations remain weak. This contrasts with the highly coherent responses of BGs and HGs, both of which rely on continuous HG networks. The heterogeneous crystalline structure of BW OGs contributes additional variability [17,18].

In GA systems (Table S3), BG penetration parameters correlated almost perfectly with HG parameters (*r* = 0.90–0.99), confirming that the HG phase is the primary determinant of BG texture. Significant BG–OG correlations were limited to *S*_B_ and *W*_10_, indicating a modest secondary contribution of the OG consistent with the discontinuous distribution of BW crystals within GA matrices [6].

In GG systems (Table S4), BG–HG correlations were also extremely high (*r* = 0.91–0.99), reflecting a rigid architecture dominated by the GG network. However, all OG parameters showed significant negative correlations with BG texture (*r* = −0.806 to −0.877), demonstrating that the OG does not reinforce the structure and may even counteract the HG response. This antagonistic effect is consistent with reports that excessive OG disrupts the continuity of GG networks [4,28].

As a whole, the OG exerts a modest reinforcing effect in GA, whereas in GG it shows a consistently negative influence, reinforcing the structural divergence between protein-based and polysaccharide-based BGs [21,25].

### 2.6. Melting Points of BGs, OGs and BW

**Figure 6:**
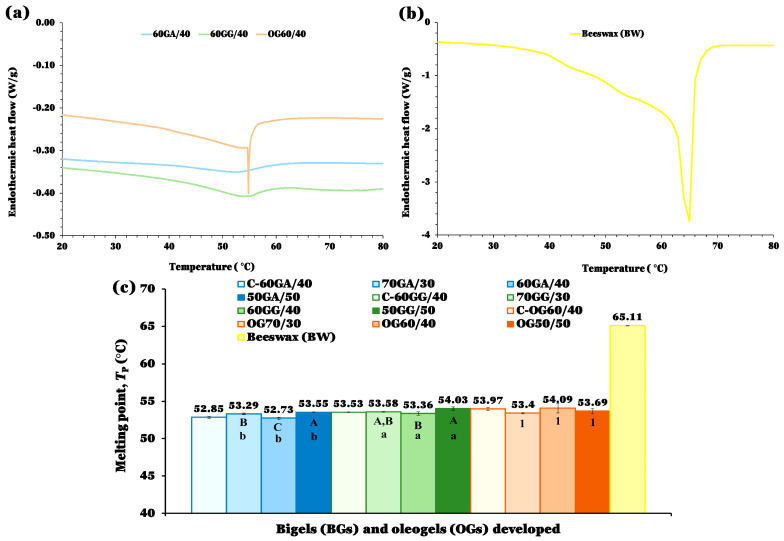
Melting profiles and peak maximum temperatures (*T*_P_) obtained by DSC: (**a**) For bigels (BGs) and individual oleogel (OG) formulated at 60/40 ratio; (**b**) For beeswax (BW); (**c**) For BGs and individual OGs formulated at 70/30, 60/40, and 50/50 ratios. ^A–C^ Effect of HG/OG ratio; for the same system (BG or HG), and for the same hydrogel-forming agent, different letters in the same column indicate significant differences (*p* < 0.05). _a,b_ Effect of hydrogel-forming agent; for the same system (BG or HG), and for the same HG/OG ratio, different letters in the same column indicate significant differences (*p* < 0.05). ^1^ Effect of HG/OG ratio in OG; different numbers in the same column indicate significant differences (*p* < 0.05).

Melting profiles of GA- and GG-based BGs at the intermediate HG/OG ratio (60/40), together with the corresponding OGs, are shown in Figure 6a, while the melting profile of pure BW is presented in Figure 6b. Similar thermal patterns were obtained for BGs and OGs at 70/30 and 50/50 ratios, as well as for the control samples. BW exhibited a single major endothermic transition at 65.11 °C, which was also detected in the individual OG phases and in BGs containing 5% BW. By contrast, this characteristic wax-associated peak was not observed in BGs due to the presence of the HG phase. Individual HGs showed no thermal transitions within the studied temperature range, confirming that all observed endothermic events originate from BW. These thermal transitions confirm the presence of BW crystalline domains prior to melting. These results agree with previous reports describing BW melting between 61 and 63 °C and, in some cases, two transitions associated with hydrocarbons (~53 °C) and wax esters (~63 °C) [17,35,36].

Melting points (*T*_P_) of BGs, OGs, and BW are summarized in Figure 6c. For BGs and OGs, *T*_P_ values ranged from 52.73 to 54.09 °C. Within each hydrogelifying agent, the 50_GA_/50 and 50_GG_/50 BGs exhibited significantly higher *T*_P_ values (*p* < 0.05) than their 60/40 and 70/30 counterparts, reflecting their higher BW content (6.25%, as described in Section 4.2). No significant differences were observed among OGs at different ratios. Across all formulations, GA-containing BGs consistently showed slightly lower *T*_P_ values than GG-based BGs. This difference likely arises from the more flexible and less rigid GA network, which allows greater mobility of dispersed BW droplets and facilitates earlier melting. Conversely, the stiffer GG network restricts droplet mobility and delays melting, resulting in marginally higher *T*_P_ values.

### 2.7. Color of BGs, HGs, and OGs

**Figure 7:**
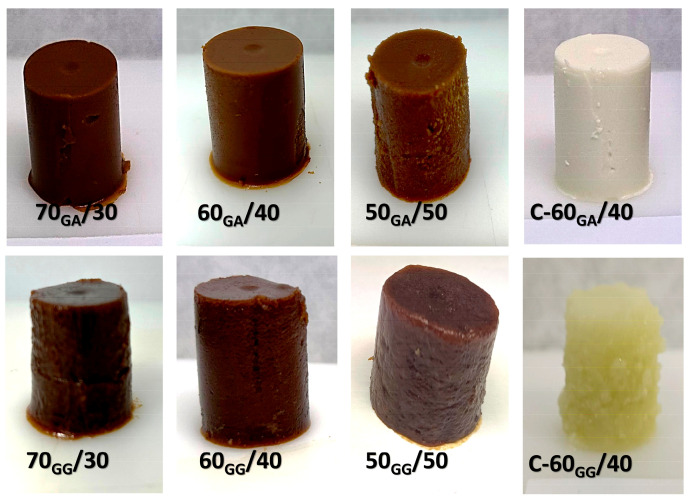
Appearance of bigels (BGs) formulated with gelatin (GA) and gellan gum (GG) as hydrogel-forming agents at 70/30, 60/40, and 50/50 HG/OG ratios (sample diameter = 25 mm; height = 35 mm).

The color parameters of BGs, HGs, and OGs were significantly affected by the HG/OG ratio and, in the case of BGs and HGs, by the HG-forming agent (Table 5; Figure 7). As previously reported [9], the intrinsic color of I-CFE and P-CFE dominated the chromatic attributes of all extract-containing samples, a trend also observed here.

In GA-based BGs, increasing the OG fraction (from 70/30 to 50/50) significantly increased L* values (*p* < 0.05) and significantly decreased both a* and b* values (*p* < 0.05), reflecting the dilution of the darker extract and the greater contribution of the lighter OG phase. GG-based BGs showed the same pattern, although with consistently lower L*, a*, and b* values than GA BGs. These differences between hydrogelators were significant at 70/30 and 60/40 (*p* < 0.05), but not at 50/50, where both systems showed similar chromatic behavior. This difference arises from the naturally yellowish hue of GG, which masks part of the extract’s red and blue tones, whereas GA is more neutral and allows the extract color to appear more intensely.

The color of the control BGs further supports this interpretation: both exhibited slightly negative a* values due to the transparency of the HGs, which lack pigments capable of shifting the signal toward the red axis. Analysis of the HGs revealed significant effects of the ratio (*p* < 0.05). In GA HGs, L* and a* values increased significantly as the OG proportion in the corresponding BGs increased (*p* < 0.05), while b* values remained negative but shifted toward less green values. These changes indicate that the chromatic response of GA HGs depends not only on the extract but also on the matrix structure and its interaction with the overall BG formulation. In GG HGs, L* values also increased significantly with the ratio (*p* < 0.05), whereas a* values decreased significantly (*p* < 0.05) and b* values shifted from slightly positive to negative values, confirming that GG does not contribute intrinsic yellowness and that color is governed primarily by extract concentration.

The HG-forming agent influenced HG color mainly through the b* coordinate: GA HGs consistently showed more negative b* values than GG HGs, with significant differences at 70/30 and 60/40 (*p* < 0.05), indicating a greener tone. Differences in a* values were less consistent, and L* values remained similar between GA and GG across ratios (no significant differences). Since OG composition was identical in GA and GG systems, no differences related to the hydrogel-forming agent were expected. As anticipated, OG color parameters corresponded to the mean values of both batches. However, the HG/OG ratio produced significant changes (*p* < 0.05): L*, a* and b* values increased progressively from OG (70/30) to OG (50/50), consistent with the higher proportion of yellowish components (P-CFE, OPO, and BW) at higher OG levels. As noted previously [9], OG color is primarily governed by P-CFE and lipid components, explaining the uniform trend observed.

### 2.8. Stability and Total Loss of BGs

The visual appearance of the BGs (Figure 7) reflected the combined influence of the HG-forming agent and the HG/OG ratio, in agreement with instrumental color measurements. GA-based BGs appeared slightly lighter than GG-based ones at the same ratio, although both displayed the characteristic brown tones imparted by the extracts. The control BGs further highlighted the intrinsic contribution of the gelling agents: the 60_GG_/40 control showed a more yellowish hue than the 60_GA_/40 control, consistent with the naturally yellowish appearance of GG and the more neutral tone of GA.

Importantly, the extract-containing BGs showed a marked visual deviation from their respective controls, appearing darker, more opaque, and more intensely colored. This difference is clearly visible in Figure 7 and arises from the strong chromatic contribution of P-CFE [9], which dominates the appearance of the biphasic structure. Such a change in visual identity may influence product applicability and consumer acceptance, depending on the intended use. In food matrices such as pâtés—where these BGs are intended to be incorporated as fat replacers—the darker and more opaque appearance may be advantageous, as pâtés typically exhibit naturally brownish or reddish tones. Conversely, in lighter emulsified products, extract concentration may require adjustment to maintain the expected visual profile. Thus, the observed chromatic deviations are not only technologically meaningful but also important for future product development.

The extract concentration used in both the HG and OG phases was selected based on preliminary formulation trials and previous reports indicating that 5–6% BW and 0.5% P-CFE provide adequate structuring, antioxidant functionality and visual uniformity without compromising gel formation. Higher extract loads led to phase separation or excessive darkening, whereas lower concentrations reduced functional performance. Thus, the chosen levels represent a balance between technological feasibility, structural integrity, and functional dose considerations.

**Figure 8:**
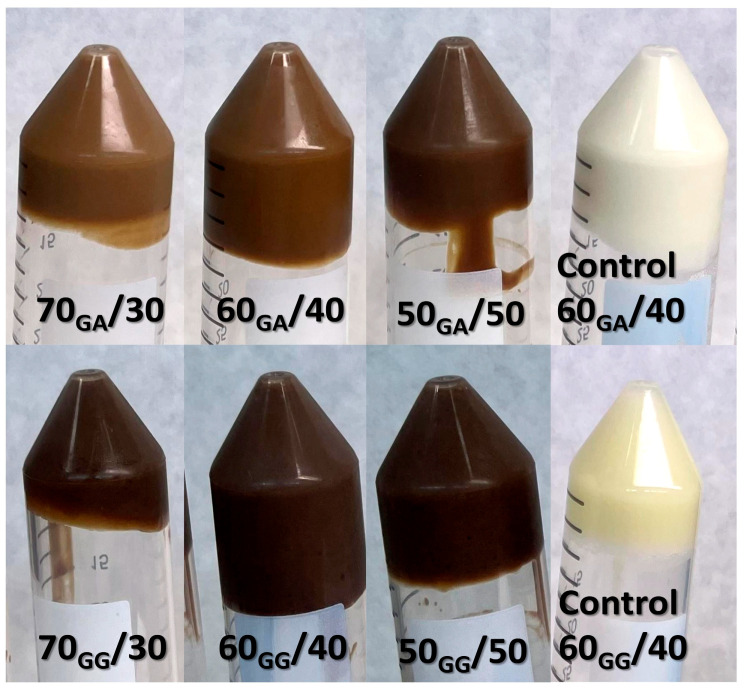
Inverted vial test images of bigels (BGs) formulated with gelatin (GA) and gellan gum (GG) as hydrogel-forming agents at 70/30, 60/40, and 50/50 HG/OG ratios on day 3 (samples placed in standard 50 mL Falcon tubes; tube diameter = 30 mm).

Physical stability was evaluated after 3 days and again after 1 month of storage. In contrast to the behavior previously reported by Álvarez et al. [9] for BGs formulated with alginate as the HG-forming agent, none of the eight formulations showed structural disruption, phase separation, or loss of self-standing ability at either time point. All bigels remained cohesive and non-flowing during the inverted vial test, demonstrating their capacity to withstand gravitational stress throughout storage. No detectable losses of aqueous or lipid phases were observed, confirming full retention within the BG network. Given that the visual appearance of the samples did not change over time, Figure 8 presents only the inverted vial test images obtained on day 3, which are representative of the behavior observed after 1 month.

In summary, the results demonstrate excellent physical stability across all formulations, regardless of the hydrogel-forming agent or HG/OG ratio.

### 2.9. Microstructure of BGs

**Figure 9:**
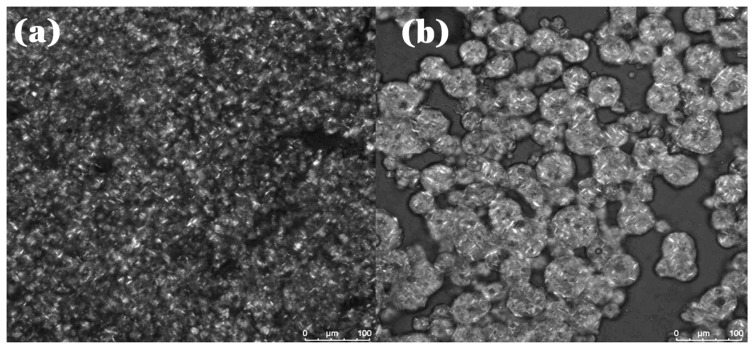
Polarized light micrographs of bigels formulated at a 60/40 HG/OG ratio: (**a**) 60_GA_/40 and (**b**) 60_GG_/40. Scale bar: 100 µm.

Representative micrographs of BGs formulated at a 60/40 HG/OG ratio are shown (Figure 9), as the microstructures observed at 50/50 and 70/30 were highly similar within each hydrogel-forming agent (GA or GG) and did not provide additional structural distinctions. In all cases, the HG constitutes the continuous phase, while the OG appears as dispersed domains in which beeswax crystals can be identified.

The 60_GA_/40 BG (Figure 9a) exhibited a fine, homogeneous, and densely packed microstructure, with small and uniformly distributed OG domains embedded within the continuous gelatin network. By contrast, the 60_GG_/40 BG (Figure 9b) displayed larger, irregular, and more sharply delineated OG domains, consistent with the formation of a rigid and crystal-reinforced matrix. Beeswax crystals were visible in both systems, although their integration into the network appeared more pronounced in GG.

These structural features align with the rheological behavior of the systems: the finer and more deformable microstructure of GA corresponds to its viscosity- and damping-dominated regime, whereas the larger, crystal-reinforced domains in GG are consistent with its stiffness-driven mechanical response.

## 3. Conclusions

This study demonstrates that GA- and GG-based BGs operate under two distinct mechanical regimes determined by the hydrogel-forming agent, the HG/OG ratio, and the distribution of carob fruit extracts. GA bigels formed soft, deformable, and viscous networks governed by hydration and energy dissipation, whereas GG BGs developed rigid, crystal-reinforced structures dominated by oscillatory stiffness. The HG/OG ratio modulated these behaviors in opposite ways: reducing water weakened GA networks, while increasing OG content strengthened GG matrices through beeswax-crystal integration.

Analyses of individual phases confirmed that HGs largely determine BG texture, with OGs providing moderate reinforcement in GA and a neutral or antagonistic effect in GG. Extract incorporation enhanced both phases, particularly GA, through protein–polyphenol interactions. Thermal and stability assessments showed narrow melting ranges (≈53–54 °C) and one-month structural stability across all formulations.

In summary, GA and GG offer complementary design spaces: GA enables soft and processable BGs suited for deformation-driven applications, while GG provides rigid and stable matrices for structures requiring high mechanical strength. These insights support the rational design of extract-loaded dual-phase BGs for functional food applications, including their future use as fat replacers in pâtés and related emulsified products. Future work will address the oxidative stability of these systems, including the evaluation of lipid oxidation markers during storage.

## 4. Materials and Methods

### 4.1. Materials

Inositol-rich carob fruit (I-CFE) and polyphenol-rich carob fruit (P-CFE) extracts were kindly provided by PlanTech BioTechnology Spain S.L. (Valencia, Spain) under the brand NOW^®^ (Nutrition Optimised Within). I-CFE is a concentrated carob syrup obtained exclusively with water as solvent. According to the technical data sheet, it contains 150 g/kg of total inositols (D-pinitol + myo-inositol), 6 g/100 g of sugars, 22.1 g/100 g of polyols, 5.1 g/100 g of dietary fiber, and 3.8 g/100 g of protein, with typical physicochemical parameters of concentrated carob pod matrices (°Brix 65–68; pH ≈ 4). P-CFE is a powdered carob extract characterized by ≥30 g/100 g of condensed tannins, ≥70 g/100 g of total dietary fiber (mostly insoluble), and low levels of polyols (1.4 g/100 g) and sugars (3 g/100 g). The extract is water-insoluble, highly astringent, and derived from carob pod material naturally enriched in polymeric tannins.

Food-grade crushed gelatin (GA) with a Bloom strength of 200/220 was supplied by Manuel Riesgo S.A. (Madrid, Spain). Gellan gum (GG), E418 food-grade, low-acyl, was purchased from Sosa Ingredients S.L. (Barcelona, Spain). Yellow beeswax (BW) pearls were provided by Iberceras Specialties Slu (Madrid, Spain). Soy lecithin (Verolec Non-GMO IP) was obtained from Lasenor Emul S.L. (Barcelona, Spain).

Refined olive pomace oil (OPO), produced in Spain, was donated by Interprofesional del Aceite de Orujo de Oliva (ORIVA, Sevilla, Spain) and refined by ACESUR S.A. (Sevilla, Spain). As established in EU Regulation No. 1308/2013 [37], refined OPO is a regulated commercial category with defined quality specifications. Its composition has been extensively characterized in Álvarez et al. [32], where it is reported to contain a fatty acid profile dominated by oleic acid (~73%), followed by palmitic (~11%) and linoleic (~10%) acids, and a triacylglycerol (TAG) distribution rich in triolein (OOO), palmitoyl-diolein (POO), and linoleoyl-diolein (LOO). These features confer high oxidative stability and technological suitability for OG structuring.

### 4.2. Preparation of I-CFE-Loaded HGs, P-CFE-Loaded OGs and BGs

HGs, OGs, and BGs were prepared at a total mass of 100 g following the compositions detailed in Table 6. I-CFE was incorporated into the HG phase and P-CFE into the OG phase in fixed amounts across all formulations. BGs were produced at HG/OG ratios of 70/30, 60/40, and 50/50 using GA- or GG-based HGs. These ratios were selected to cover the transition from HG-dominated systems (70/30) to more structurally balanced matrices (60/40) and OG-reinforced networks (50/50), while maintaining food-relevant textures and workable biphasic structures. Control formulations were prepared only at the 60/40 ratio, as preliminary trials showed that this proportion provides the most representative balance between phases for isolating the specific effect of the carob fruit extracts.

HG preparation: GA-based HGs were prepared by dispersing the required amounts of GA and water (Table 6) and heating to ~60 °C until complete dissolution. GG-based HGs were prepared similarly but heated to ~90 °C. Once the gelling agent was fully dissolved, I-CFE was added, and the HG was maintained in a water bath for ~12 min to ensure temperature equilibration before mixing. In GG systems, I-CFE was incorporated only after the dissolution step and subsequent temperature equilibration, minimizing exposure of the extract to elevated thermal loads.

OG preparation: OGs were prepared by heating OPO, BW, and P-CFE (Table 1) to 70 °C under magnetic stirring (400 rpm) until complete melting. Soy lecithin was then added, and the mixture was homogenized with an Ultra-Turrax at 9000 rpm for 1 min before equilibrating its temperature with that of the HG. This temperature ensured complete melting of the lipid structurant while avoiding exposure of P-CFE to the higher thermal loads required for HG dissolution.

BG preparation: Once both phases reached similar temperatures, the HG was homogenized for 1 min at 9000 rpm. The OG was then added over the HG while homogenizing for 2 min, followed by a final 1-min homogenization to obtain the BG structure. BGs were transferred into sterile containers and stored at 4 °C for 72 h to allow complete gel setting before analysis.

### 4.3. Rheological Measurements of BGs

Rheological measurements were performed using a Kinexus Pro controlled-stress rheometer (NETZSCH-Gerätebau GmbH, Wittelsbacherstr., Selb, Germany) at 25 °C. A sandblasted parallel-plate geometry was used, consisting of an upper PU20 L5272 plate (20 mm diameter) and a 1.5 mm gap, as described previously [9]. Disc-shaped BG samples were placed on the sandblasted lower plate and allowed to rest for 5 min prior to testing to ensure thermal and mechanical equilibration. A plastic solvent trap was used throughout the measurements to minimize evaporation. Temperature was controlled to within ±0.1 °C using an environmental cartridge. All measurements were performed in triplicate for each BG formulation.

#### 4.3.1. Small Amplitude Oscillatory Shear (SAOS) Measurements

##### Stress Sweep Tests

The linear viscoelastic region (LVR) of each BG sample was determined through stress sweep tests performed at a constant frequency of 1 Hz. When GA was used as the gelling agent, the applied shear stress (*σ*) ranged from 1 to 100 Pa for the 50/50 ratio and from 0.5 to 50 Pa for the 60/40 and 70/30 ratios. When GG was used, *σ* ranged from 1 to 100 Pa for the 70/30 ratio, from 2 to 200 Pa for the 60/40 ratio, and from 3 to 300 Pa for the 50/50 ratio. For the control samples prepared at a 60/40 ratio and without extracts in the individual phases, *σ* ranged from 0.5 to 50 Pa for GA and from 2 to 200 Pa for GG. A total of 41 data points were collected. Variations in storage modulus (*G*′), loss modulus (*G*″), loss factor (tan *δ* = *G*″/*G*′), and complex modulus ($G*=\;\sqrt{G^{\prime 2}+G^{\prime \prime 2}}$) were monitored. Critical stress amplitude (*σ*_max_), strain amplitude (*γ*_max_), and loss factor (tan *δ*_max_) values were determined based on the G* trend, considering a ±10% tolerance range [9,38].

Additionally, differences in the LVR were assessed by fitting a linear regression between experimental σ* and shear strain (*γ*) for the G* values, from initial *σ*_0_ to *σ*_max_ and *γ*_0_ to *γ*_max_ (Equation (1)), to obtain coefficients “*a*” and “*b*”.

σ* = *a*·*γ* + *b* (1)

The slope *a* (Pa) reflects the overall resistance to deformation (including elastic and viscous contributions) and is considered equivalent to gel strength [39,40] (Borderías et al., 2020; Montero et al., 2024). The intercept *b* (Pa) represents the initial stress (*σ*_0_ at *γ*_0_). Both parameters were subsequently used to calculate the energy term *E* (Equation (2)), which quantifies the total energy or toughness involved in the linear deformation of the BG network [38,41].

(2) $$E=\int_{\gamma_{0}}^{\gamma_{max}}\left(a\cdot \gamma +b\right)\cdot d\gamma$$

##### Frequency Sweep Tests

BGs were also subjected to harmonic strain oscillations at varying frequencies, using a fixed shear stress amplitude (*σ*) to ensure that the strain remained within the LVR. When GA was used as the gelling agent, *σ* was set to 1 Pa for the 70/30 ratio and to 5 Pa for the 50/50 and 60/40 ratios. When GG was used, *σ* was set to 5 Pa for the 70/30 ratio and to 10 Pa for the 60/40 and 50/50 ratios. For the control samples without extracts at the 60/40 ratio, *σ* was 1 Pa for GA and 10 Pa for GG. Frequency sweep tests were conducted from 10 to 0.1 Hz, and *G*′, *G*″, tan *δ*, and complex viscosity (η* = G*/*ω*; where *ω* is the angular frequency in rad·s^−1^) were determined as functions of frequency. In addition, G* was fitted to the power-law relationship known as the weak gel model, as described by Gabriele et al. [31] (Equation (3)):

G*(*f*) = *Af*^1/z^ (3)

where G* is the complex modulus (Pa); *f* is the frequency (Hz); *A* (Pa·s^1/z^) is the proportionality constant representing the strength of the interactions (G* at 1 Hz); and *z* (dimensionless) is the coordination number or network connectivity [32], which can be considered as indicator of structural organization.

#### 4.3.2. Steady Shear Measurements

##### Flow Behavior

For each BG sample, the flow curve was obtained as a function of shear rate, ranging from 10 to 0.1 s^−1^, with 10 measurement points per decade. The influence of shear rate on apparent viscosity was described using the power-law model (Equation (4)):

(4) $$\eta \left(\dot{\gamma }\right)=K{\dot{\gamma }}^{n-1}$$

where *η* is the apparent viscosity (Pa s); $\dot{\gamma }$ is the shear rate (s^−1^); *K* is the consistency index (Pa·s^*n*^), which correspond to the apparent viscosity at 1 s^−1^; and *n* is the flow behavior index (dimensionless), indicating the degree of deviation from Newtonian behavior. Apparent viscosities (Pa·s) at fixed shear rates of 10 s^−1^—representative of oral shear conditions [42]—and 0.1 s^−1^—representative of low-shear processes—were also evaluated for the BGs.

##### Three-Step Shear Rate Tests

For viscometric rebuild analysis, samples were first subjected to a shear rate of 0.1 s^−1^ for 30 s. In the second stage, the shear rate was increased to 10 s^−1^ and applied for 30 s to simulate structural breakdown. Finally, in the third stage, the shear rate was reduced again to 0.1 s^−1^, and viscosity recovery was monitored for 600 s. The percentage of viscosity recovery at the end of the test was calculated by comparing the final viscosity values after the two low shear rate steps [29].

### 4.4. Texture Measurements of BGs, HGs and OGs

Penetration tests were performed using a Texture Analyzer (TA.HDPlus, Stable Micro Systems Ltd., Godalming, UK) equipped with a 5 kg load cell and operated via Texture Exponent software (version 6,2,6,0). Measurements were conducted at 5 ± 1 °C using a 2 mm cylindrical flat stainless-steel probe (P/2; Stable Micro Systems Ltd., Godalming, UK). The probe penetrated the sample to a depth of 10 mm at a constant speed of 3 mm s^−1^, as described previously [43]. From the resulting force–distance curves, the following parameters were obtained: force at 10 mm (*F*_10_, N); total work up to 10 mm (*W*_10_, mJ); and, at the first rupture peak, the breaking force (*F*_B_, N) and breaking slope (*S*_B_, N mm^−1^). These four parameters are commonly interpreted as related indices of hardness (*F*_10_), work of penetration (*W*_10_), penetration or breaking force (*F*_B_), and cohesiveness or structural integrity (*S*_B_), respectively. All measurements were performed at least in triplicate.

### 4.5. Thermal Behavior of BGs, HGs and OGs

A TA Q1000 differential scanning calorimeter (TA Instruments, New Castle, DE, USA) was used to evaluate the thermal characteristics of the different systems. A 15–20 mg amount of each sample was placed in an aluminum pan and hermetically sealed. An empty pan was used as a reference. For heating thermograms, the samples were heated from 0 to 100 °C with a constant heating rate of 5 °C/min, as described previously [9]. The peak maximum temperature (*T*_P_, °C) or melting point was recorded for all major melting peaks of the BGs and the individual OG phases, attributed to the BW portions of the OGs, using Universal Analysis 2000. Samples were always evaluated in triplicate.

### 4.6. Color of BGs, HGs, and OGs

Color characterization of the BGs, HG, and OGs was conducted using a Konica Minolta CM-3500D spectrophotometer (Konica Minolta Business Technologies, Tokyo, Japan). The instrument was calibrated before each measurement session using the standard white calibration tile provided by the manufacturer. The spectrophotometer was positioned on a glass plate and operated with a D65 illuminant and a 10° standard observer. The recorded parameters included lightness (L*), redness/greenness (±a*), and yellowness/blueness (±b*). Each sample was analyzed in ten independent readings.

### 4.7. Microstructural Analysis of BGs

The microstructure of the bigels was examined via polarized light microscopy (PLM), following the general procedure described by Álvarez et al. [9], with minor adaptations. Approximately 15 mg of each refrigerated BG sample were placed on a glass slide, covered with a coverslip and stored at 4 °C for 72 h to promote cooling and beeswax recrystallization prior to imaging. Micrographs were acquired using a Leica AF6000 LX microscope (Mannheim, Germany), equipped with a Hamamatsu C9100-02 digital camera (Hamamatsu, Japan) and maintained within a temperature-controlled chamber at 37 °C. Observations were performed using 10×/0.30 NA and 40×/0.75 NA objectives, with an additional 1.6× magnification and illumination provided by an Hg-arc lamp. Images were recorded at 1000 × 1000 px resolution using LAS X software (v.5.3.1, Leica Microsystems).

### 4.8. Statistical Analysis

All statistical analyses were performed using SPSS Statistics v.30 (IBM Corp., Armonk, NY, USA). A two-factor ANOVA was first applied to evaluate the effects of (i) the HG/OG ratio (70/30, 60/40, 50/50) and (ii) the hydrogelifying agent (GA or GG) on all rheological (SAOS and steady shear), textural, thermal, and color parameters. Because significant interactions were observed for all variables (*p* < 0.05), data were subsequently analyzed using one-way ANOVAs to isolate simple effects. Specifically, for each hydrogelifying agent, a one-way ANOVA assessed the influence of the HG/OG ratio, whereas for each ratio, a one-way ANOVA evaluated the effect of the gelling agent (GA vs. GG). Tukey’s HSD test was applied when significant differences were detected (*p* < 0.05).

To explore multivariate patterns and identify dominant mechanical domains, two categorical principal components analyses (CATPCA) were performed. CATPCA is an extension of principal component analysis that incorporates optimal scaling to jointly analyze numerical and categorical variables. The first included all rheological and textural parameters of the BGs, while the second incorporated the textural attributes of BGs, HGs, and OGs. Model suitability was assessed through total variance explained and Cronbach’s *α*.

Pearson correlation analyses were conducted separately for GA and GG systems to examine internal mechanical architecture. Correlations were computed for oscillatory, steady-shear, and textural variables of BGs. Additional matrices evaluated relationships among BG, HG, and OG textural parameters within each gelling system. Statistical significance was set at *p* < 0.05.

## Figures and Tables

**Table 1:** Critical rheological parameters defining the linear viscoelastic region (LVR) at 25 °C for formulated bigels (BGs).

BG	*σ*_max_ (Pa)	*γ*_max_ (%)	tan *δ*_max_ (−)	*a* (Pa)	*b* (Pa)	*R*^2^ (Equation (1))	*E* (J/m^3^)
C-60_GA_/40	4.02 ± 0.004	0.099 ± 0.008	0.139 ± 0.001	4028 ± 122.5	9.49 ± 1.26	1.00	96.73 ± 2.18
70_GA_/30	7.14 ± 0.015 ^B^_b_	0.154 ± 0.032 ^B^_b_	0.176 ± 0.005 ^C^_b_	4832 ± 43.05 ^C^_b_	11.44 ± 1.07^C^_b_	0.999	267.71 ± 23.81 ^B^_b_
60_GA_/40	12.66 ± 0.010 ^A^_b_	0.217 ± 0.012 ^A^_a_	0.229 ± 0.004 ^B^_b_	5977 ± 33.40 ^B^_b_	24.70 ± 1.64 ^A^_b_	0.997	650.57 ± 15.89 ^A^_a_
50_GA_/50	6.33 ± 0.000 ^C^_b_	0.051 ± 0.001 ^C^_a_	0.305 ± 0.008 ^A^_a_	12,165 ± 196.52 ^A^_b_	18.32 ± 0.212 ^B^_b_	0.999	78.36 ± 1.43 ^C^_b_
C-60_GG_/40	22.45 ± 0.000	0.022 ± 0.002	0.301 ± 0.011	108,865 ± 9157	61.55 ± 7.00	0.997	121.3± 3.29
70_GG_/30	14.23 ± 0.005 ^C^_a_	0.213 ± 0.011 ^A^_a_	0.340 ± 0.008 ^A^_a_	6743 ± 217.3 ^C^_a_	35.45 ± 0.628 ^C^_a_	0.998	725.0 ± 34.26 ^A^_a_
60_GG_/40	22.46 ± 0.000 ^B^_a_	0.042 ± 0.002 ^B^_b_	0.347 ± 0.018 ^A^_a_	54,377 ± 4308 ^B^_a_	48.99 ± 1.57 ^B^_a_	0.999	226.6 ± 3.72 ^C^_b_
50_GG_/50	30.02 ± 0.010 ^A^_a_	0.043 ± 0.002 ^B^_b_	0.323 ± 0.014 ^A^_a_	75,781 ± 861.5 ^A^_a_	67.74 ± 6.33 ^A^_a_	0.999	317.6 ± 20.57 ^B^_a_

Mean value (*n* = 3) ± standard deviation. *σ*_max_, critical value of shear stress; *γ*_max_, critical value of shear strain; tan *δ*, critical value of the loss factor (tan *δ* = *G*″/*G*′); *a*, gel strength or total resistance (elastic and viscous) to deformation; *b*, stress at the initial state (*σ*_0_); *R*^2^, determination coefficient of linear regression fit of stress (*σ*) versus strain (*γ*) for the complex modulus (G*); *E*, area under straight line of *σ*_max_ versus *γ*_max_ from Equation (2). ^A–C^ Effect of HG/OG ratio; for the same hydrogel-forming agent, different letters in the same column indicate significant differences (*p* < 0.05). _a,b_ Effect of hydrogel-forming agent; for the same HG/OG ratio, different letters in the same column indicate significant differences (*p* < 0.05).

**Table 2:** Mechanical spectra data at 1 Hz and at 25 °C, and weak gel model parameters for formulated bigels (BGs).

BG	*G*′ (Pa)	*G*″ (Pa)	tan *δ* (−)	η* (Pa·s)	*A* (Pa·s^1/^*^z^*)	*z* (−)	*R*^2^ (Equation (3))
C-60_GA_/40	4310 ± 222.0	590.0 ± 34.52	0.137 ± 0.001	692.2 ± 35.72	4385 ± 214.5	11.95 ± 0.953	0.994
70_GA_/30	4284 ± 257.7 ^C^_b_	686.3 ± 46.98 ^C^_b_	0.160 ± 0.001 ^C^_b_	690.4 ± 41.65 ^C^_b_	4332 ± 242.1 ^C^_b_	10.48 ± 0.364 ^A^_a_	0.996
60_GA_/40	6445 ± 302.6 ^B^_b_	1370 ± 57.46 ^B^_b_	0.213 ± 0.002 ^B^_b_	1049 ± 48.85 ^B^_b_	6593 ± 222.3 ^B^_b_	8.04 ± 0.528 ^B^_a_	0.997
50_GA_/50	11,230 ± 113.6 ^A^_b_	3453 ± 130.1 ^A^_b_	0.307 ± 0.011 ^A^_a_	1870 ± 20.79 ^A^_b_	11,978 ± 115.7 ^A^_b_	5.45 ± 0.340 ^C^_a_	0.996
C-60_GG_/40	80,337 ± 5341	23,243 ± 313.7	0.290 ± 0.019	13,313 ± 819.9	85,939 ± 4805	5.90 ± 0.194	0.984
70_GG_/30	6983 ± 248.0 ^C^_a_	2295 ± 23.03 ^C^_a_	0.329 ± 0.009 ^A^_a_	1170 ± 38.74 ^C^_a_	7286 ± 236.2 ^C^_a_	5.26 ± 0.025 ^B^_b_	0.998
60_GG_/40	57,590 ± 2160 ^B^_a_	18,633 ± 96.09 ^B^_a_	0.324 ± 0.013 ^A^_a_	9634 ± 324.1 ^B^_a_	60,212 ± 1793 ^B^_a_	5.19 ± 0.048 ^B^_b_	1.00
50_GG_/50	95,247 ± 4359 ^A^_a_	28,677 ± 223.68 ^A^_a_	0.302 ± 0.016 ^A^_a_	15,830 ± 656.0 ^A^_a_	102,323 ± 4168 ^A^_a_	5.69 ± 0.046 ^A^_a_	0.989

Mean value (*n* = 3) ± standard deviation. *G*′, storage modulus; *G*″, loss modulus; tan *δ*, loss factor; η*, complex viscosity; *A*, interaction strength; *z*, network extension; *R*^2^, determination coefficient for Equation (3). ^A–C^ Effect of HG/OG ratio; for the same hydrogel-forming agent, different letters in the same column indicate significant differences (*p* < 0.05). _a,b_ Effect of hydrogel-forming agent; for the same HG/OG ratio, different letters in the same column indicate significant differences (*p* < 0.05).

**Table 3:** Steady-shear rheological properties at 25 °C for formulated bigels (BGs).

BG	*η*_0.1_ (Pa·s)	*η*_10_(Pa·s)	*K* (Pa·s*^n^*)	*n* (−)	*R*^2^ (Equation (4))	Viscosity Recovery (%)
C-60_GA_/40	1993 ± 22.00	78.70 ± 1.61	321.8 ± 1.71	0.272 ± 0.001	0.994	33.69 ± 0.552
70_GA_/30	3963 ± 71.00 ^B^_a_	86.88 ± 3.06 ^A^_a_	451.6 ± 36.74 ^B^_a_	0.146 ± 0.016 ^B^_b_	0.991	53.58 ± 3.45 ^A^_a_
60_GA_/40	4949 ± 81.50 ^A^_a_	85.65 ± 2.60 ^A^_a_	540.5 ± 39.09 ^A^_a_	0.097 ± 0.004 ^C^_b_	0.996	55.37 ± 4.92 ^A^_a_
50_GA_/50	915.5 ± 64.45 ^C^_a_	22.79 ± 1.90 ^B^_a_	120.9 ± 9.11 ^C^_a_	0.173 ± 0.002 ^A^_a_	0.997	22.97 ± 1.11 ^B^_b_
C-60_GG_/40	177.3 ± 4.00	7.38 ± 0.414	31.50 ± 2.17	0.285 ± 0.016	0.997	32.26 ± 1.98
70_GG_/30	657.6 ± 37.45 ^A^_b_	25.17 ± 1.70 ^A^_b_	112.5 ± 1.59 ^A^_b_	0.280 ± 0.001 ^A^_a_	0.996	37.99 ± 2.04 ^A^_b_
60_GG_/40	384.9 ± 2.50 ^C^_b_	12.33 ± 0.545 ^B^_b_	61.14 ± 1.79 ^C^_b_	0.240 ± 0.027 ^A^_a_	0.997	25.26 ± 0.410 ^C^_b_
50_GG_/50	569.0 ± 26.50 ^B^_b_	14.05 ± 0.200 ^B^_b_	78.46 ± 2.09 ^B^_b_	0.168 ± 0.008 ^B^_a_	0.995	31.42 ± 1.62 ^B^_a_

Mean value (*n* = 3) ± standard deviation. *η*_0.1_ and *η*_10_, apparent viscosities measured at fixed shear rates of 0.1 and 10 s^−1^, respectively; *K*, consistency index; *n*, flow behavior index; *R*^2^, determination coefficient for Equation (4). ^A–C^ Effect of HG/OG ratio; for the same hydrogel-forming agent, different letters in the same column indicate significant differences (*p* < 0.05). _a,b_ Effect of hydrogel-forming agent; for the same HG/OG ratio, different letters in the same column indicate significant differences (*p* < 0.05).

**Table 4:** Penetration parameters at 5 °C for formulated bigels (BGs), hydrogels (HGs), and oleogels (OGs).

BG	*F*_10_ (N)	*W*_10_ (mJ)	*F*_B_ (N)	*S*_B_ (N mm^−1^)
C-60_GA_/40	0.420 ± 0.008	3.20 ± 0.048	0.285 ± 0.008	0.151 ± 0.005
70_GA_/30	0.501 ± 0.039 ^A^_a_	4.04 ± 0.061 ^A^_a_	0.546 ± 0.006 ^A^_a_	0.216 ± 0.009 ^A^_a_
60_GA_/40	0.459 ± 0.026 ^A^_a_	3.84 ± 0.022 ^B^_a_	0.492 ± 0.001 ^B^_a_	0.199 ± 0.003 ^A^_a_
50_GA_/50	0.346 ± 0.007 ^B^_a_	2.75 ± 0.055 ^C^_a_	0.278 ± 0.006 ^C^_a_	0.140 ± 0.005 ^B^_b_
C-60_GG_/40	0.134 ± 0.005	1.30 ± 0.059	0.097 ± 0.004	0.113 ± 0.019
70_GG_/30	0.084 ± 0.002 ^C^_b_	0.736 ± 0.008 ^C^_b_	0.082 ± 0.002 ^C^_b_	0.113 ± 0.003 ^C^_b_
60_GG_/40	0.181 ± 0.003 ^B^_b_	1.53 ± 0.055 ^B^_b_	0.140 ± 0.004 ^B^_b_	0.149 ± 0.004 ^B^_b_
50_GG_/50	0.327 ± 0.012 ^A^_a_	2.58 ± 0.107 ^A^_a_	0.230 ± 0.010 ^A^_b_	0.219 ± 0.006 ^A^_a_
C-HG_60GA/40_	0.418 ± 0.013	3.50 ± 0.028	0.598 ± 0.021	0.147 ± 0.002
HG_70GA/30_	0.739 ± 0.034 ^A^_a_	6.20 ± 0.110 ^A^_a_	1.47 ± 0.006 ^A^_a_	0.237 ± 0.003 ^A^_a_
HG_60GA/40_	0.765 ± 0.039 ^A^_a_	5.60 ± 0.148 ^B^_a_	1.09 ± 0.029 ^B^_a_	0.207 ± 0.002 ^B^_b_
HG_50GA/50_	0.387 ± 0.013 ^B^_a_	2.41 ± 0.140 ^C^_a_	0.305 ± 0.013 ^C^_a_	0.084 ± 0.004 ^C^_b_
C-HG_60GG/40_	0.016 ± 0.001	0.137 ± 0.010	0.031 ± 0.000	0.222 ± 0.007
HG_70GG/30_	0.049 ± 0.003 ^B^_b_	0.479 ± 0.007 ^C^_b_	0.073 ± 0.003 ^C^_b_	0.068 ± 0.002 ^C^_b_
HG_60GG/40_	0.089 ± 0.006 ^B^_b_	0.983 ± 0.012 ^B^_b_	0.210 ± 0.010 ^B^_b_	0.256 ± 0.008 ^B^_a_
HG_50GG/50_	0.240 ± 0.041 ^A^_b_	1.48 ± 0.211 ^A^_b_	0.333 ± 0.007 ^A^_a_	0.340 ± 0.007 ^A^_a_
C-OG_60/40_	0.306 ± 0.010	2.40 ± 0.066	0.318 ± 0.023	1.41 ± 0.057
OG_70/30_	0.704 ± 0.023 ^1^	5.85 ± 0.217 ^1^	0.578 ± 0.048 ^2^	1.53 ± 0.089 ^2^
OG_60/40_	0.547 ± 0.030 ^2^	5.29 ± 0.179 ^1,2^	0.722 ± 0.027 ^1^	1.97 ± 0.115 ^1^
OG_50/50_	0.581 ± 0.027 ^2^	4.90 ± 0.279 ^2^	0.599 ± 0.012 ^2^	0.821 ± 0.044 ^3^

Mean value (*n* = 3) ± standard deviation. *F*_10_, force at 10 mm; *W*_10_, total work up to 10 mm; *F*_B_ and *S*_B_, breaking force and slope at the first rupture peak, respectively. ^A–C^ Effect of HG/OG ratio; for the same system (BG or HG), and for the same hydrogel-forming agent, different letters in the same column indicate significant differences (*p* < 0.05). _a,b_ Effect of hydrogel-forming agent; for the same system (BG or HG), and for the same HG/OG ratio, different letters in the same column indicate significant differences (*p* < 0.05). ^1−3^ Effect of HG/OG ratio in OG; different numbers in the same column indicate significant differences (*p* < 0.05).

**Table 5:** Color parameters for formulated bigels (BGs), hydrogels (HGs), and oleogels (OGs).

BG	L*	a*	b*
C-60_GA_/40	86.13 ± 0.552	−3.76 ± 0.096	12.07 ± 0.266
70_GA_/30	32.77 ± 0.217 ^B^_a_	9.81 ± 0.081 ^A^_a_	6.40 ± 0.101 ^A^_a_
60_GA_/40	30.42 ± 0.220 ^C^_a_	8.85 ± 0.152 ^B^_a_	3.30 ± 0.108 ^B^_a_
50_GA_/50	35.08 ± 0.994 ^A^_a_	9.73 ± 0.305 ^A^_a_	0.514 ± 0.088 ^C^_b_
C-60_GG_/40	62.82 ± 0.281	−4.40 ± 0.102	11.06 ± 0.260
70_GG_/30	27.65 ± 0.160 ^C^_b_	7.05 ± 0.091 ^B^_b_	3.79 ± 0.068 ^A^_b_
60_GG_/40	29.59 ± 0.651 ^B^_b_	7.80 ± 0.204 ^A^_b_	3.41 ± 0.197 ^B^_a_
50_GG_/50	31.72 ± 0.494 ^A^_b_	6.83 ± 0.230 ^C^_b_	2.90 ± 0.270 ^C^_a_
C-HG_60GA/40_	70.61 ± 0.376	0.593 ± 0.017	0.867 ± 0.049
HG_70GA/30_	24.50 ± 0.729 ^B^_b_	3.19 ± 0.174 ^C^_b_	−3.25 ± 0.120 ^B^_b_
HG_60GA/40_	25.38 ± 1.19 ^B^_a_	3.77 ± 0.284 ^B^_a_	−3.58 ± 0.242 ^C^_b_
HG_50GA/50_	28.21 ± 0.936 ^A^_a_	4.21 ± 0.296 ^A^_a_	−2.97 ± 0.179 ^A^_b_
C-HG_60GG/40_	73.60 ± 0.907	1.34 ± 0.021	−3.27 ± 0.097
HG_70GG/30_	25.34 ± 0.617 ^C^_a_	4.15 ± 0.294 ^A^_a_	0.176 ± 0.016 ^A^_a_
HG_60GG/40_	26.41 ± 1.17 ^B^_a_	3.97 ± 0.307 ^A^_a_	−1.71 ± 0.152 ^C^_a_
HG_50GG/50_	27.39 ± 0.647 ^A^_b_	3.13 ± 0.116 ^B^_b_	−1.29 ± 0.083 ^B^_a_
C-OG_60/40_	50.14 ± 0.416	−4.21 ± 0.098	10.22 ± 0.159
OG_70/30_	33.37 ± 0.208 ^3^	5.29 ± 0.082 ^2^	3.83 ± 0.095 ^3^
OG_60/40_	36.48 ± 0.450 ^2^	3.90 ± 0.380 ^3^	4.57 ± 0.891 ^2^
OG_50/50_	40.04 ± 0.249 ^1^	7.08 ± 0.230 ^1^	5.93 ± 0.242 ^1^

Mean value (*n* = 10) ± standard deviation. L*, a*, b*, CIELab color parameters corresponding to lightness, redness/greenness and yellowness/blueness, respectively. ^A–C^ Effect of HG/OG ratio; for the same system (BG or HG), and for the same hydrogel-forming agent, different letters in the same column indicate significant differences (*p* < 0.05). _a,b_ Effect of hydrogel-forming agent; for the same system (BG or HG), and for the same HG/OG ratio, different letters in the same column indicate significant differences (*p* < 0.05). ^1−3^ Effect of HG/OG ratio in OG; different numbers in the same column indicate significant differences (*p* < 0.05).

**Table 6:** Percentages of the different ingredients in 100 g of BG, depending on the HG-forming agent and the formulated HG/OG ratio.

	HG Phase				OG Phase			
BG	I-CFE	Water	Gelatin (GA)	Gellan Gum (GG)	P-CFE	OPO	BW	Soy Lecithin
70_GA_/30	30.00	35.00	5.00	-	1.00	25.20	3.75	0.075
60_GA_/40	30.00	25.71	4.29	-	1.00	33.90	5.00	0.100
50_GA_/50	30.00	16.43	3.57	-	1.00	42.62	6.25	0.125
70_GG_/30	30.00	39.30	-	0.70	1.00	25.20	3.75	0.075
60_GG_/40	30.00	29.40	-	0.60	1.00	33.90	5.00	0.100
50_GG_/50	30.00	19.50	-	0.50	1.00	42.62	6.25	0.125
C-60_GA_/40	-	55.71	4.29	-	-	34.90	5.00	0.100
C-60_GG_/40	-	59.40	-	0.60	-	34.90	5.00	0.100

HG, hydrogel; OG, oleogel; BG, bigel; I-CFE, inositol-rich carob fruit extract; P-CFE, phenolic compound-rich carob fruit extract; OPO, olive pomace oil; BW, beeswax.

## Data Availability

All the raw data for this article, used in the generation of tables and figures, are available at the public repository digital.csic.es of the Spanish Research Council (CSIC) at https://doi.org/10.20350/digitalCSIC/18342.

## Supplementary Materials

The following supporting information can be downloaded at: https://www.mdpi.com/article/10.3390/gels12070602/s1, Table S1: Cross-test correlations obtained from bigels (BGs) formulated with gelatin (GA) (*n* = 9) between rheological and mechanical properties and parameters derived from SAOS, steady-state, and penetration measurements; Table S2: Cross-test correlations obtained from bigels (BGs) formulated with gellan gum (GG) (*n* = 9) between rheological and mechanical properties and parameters derived from SAOS, steady-state, and penetration measurements; Table S3: Correlations obtained between penetration measurements of bigels (BGs) and hydrogels (HGs) formulated with gelatin (GA) and beeswax-based oleogels (OGs) (*n* = 9); Table S4: Correlations obtained between penetration measurements of bigels (BGs) and hydrogels (HGs) formulated with gelatin (GG) and beeswax-based oleogels (OGs) (*n* = 9).
