# Supplementary material for: Mechanical Regimes in Gelatin and Gellan Gum Bigels: Structure–Function Relationships and Dual Delivery of Carob Fruit Extracts
Source: Gels. 2026 Jul 7;12(7):602. doi: 10.3390/gels12070602 (PMC13407645; doi:10.3390/gels12070602)
Supplement: Supplementary file 1 [file gels-12-00602-s001.zip › gels-4399603-supplementary.pdf]

Table S1. Cross-test correlations obtained from bigels (BGs) formulated with gelatin (GA) (n = 9) between rheological and mechanical properties and parameters derived from SAOS, steady-state, and penetration measurements. Variables from the same test are indicated in the same color.

	σ_{max}	γ_{max}	tan δ_{max}	a	b	E	G'	G''	η^*	tan δ	A	z	η_{10}	$\eta_{0.1}$	n	K	Recovery	F_{10}	F_B	S_B	W_{10}
σ_{max}	**1**	**0.832****	-0.221	-0.475	0.778*	**0.976****	-0.325	-0.390	-0.333	-0.280	-0.341	0.130	0.583	0.767*	**-0.944****	0.739*	0.625	0.342	0.435	0.406	0.472
γ_{max}		**1**	-0.687*	**-0.838****	0.372	**0.931****	-0.750*	-0.789*	-0.755*	-0.716*	-0.761*	0.618	**0.900****	**0.963****	**-0.944****	**0.959****	**0.938****	0.761*	**0.816****	0.762*	**0.831****
tan δ_{max}			**1**	**0.961****	0.407	-0.425	**0.986****	**0.975****	**0.985****	**0.987****	**0.985****	**-0.964****	**-0.913****	-0.790*	0.440	**-0.801****	**-0.886****	**-0.918****	**-0.971****	**-0.952****	**-0.954****
a				**1**	0.164	-0.650	**0.983****	**0.992****	**0.985****	**0.972****	**0.987****	**-0.907****	**-0.988****	**-0.927****	0.648	**-0.929****	**-0.964****	**-0.915****	**-0.998****	**-.980****	**-.996****
b					**1**	0.639	0.322	0.254	0.314	0.359	0.305	-0.479	-0.039	0.207	-0.606	0.170	0.058	-0.217	-0.207	-0.232	-0.168
E						**1**	-0.517	-0.575	-0.524	-0.476	-0.532	0.336	0.741*	**0.884****	**-0.978****	**0.862****	0.780*	0.524	0.615	0.580	0.645
G'							**1**	**0.996****	**1.000****	**0.993****	**1.000****	**-0.965****	**-0.957****	**-0.855****	0.533	**-0.870****	**-0.914****	**-0.909****	**-0.990****	**-0.967****	**-0.979****
G''								**1**	**0.997****	**0.992****	**0.998****	**-0.946****	**-0.974****	**-0.889****	0.582	**-0.898****	**-0.936****	**-0.916****	**-0.995****	**-0.981****	**-0.988****
η^*									**1**	**0.993****	**1.000****	**-0.963****	**-0.960****	**-0.860****	0.539	**-0.873****	**-0.917****	**-0.910****	**-0.991****	**-0.969****	**-0.980****
tan δ										**1**	**0.994****	**-0.966****	**-0.939****	**-0.828****	0.483	**-0.838****	**-0.899****	**-0.914****	**-0.980****	**-0.976****	**-0.967****
A											**1**	**-0.960****	**-0.962****	**-0.864****	0.545	**-0.877****	**-0.922****	**-0.914****	**-0.993****	**-0.972****	**-0.983****
z												**1**	**0.868****	0.723*	-0.379	0.753*	**0.813****	**0.839****	**0.925****	**0.891****	**0.892****
η_{10}													**1**	**0.968****	-0.748*	**0.973****	**0.975****	**0.891****	**0.981****	**0.954****	**0.984****
$\eta_{0.1}$														**1**	**-0.876****	**0.993****	**0.962****	**0.809****	**0.909****	**0.882****	**0.924****
n															**1**	**-0.876****	-0.778*	-0.533	-0.618	-0.552	-0.641
K																**1**	**0.954****	**0.808****	**0.914****	**0.873****	**0.925****
Recovery																	**1**	**0.907****	**0.953****	**0.922****	**0.957****
F_{10}																		**1**	**0.908****	**0.909****	**0.929****
F_B																			**1**	**0.978****	**0.993****
S_B																				**1**	**0.979****
W_{10}																					**1**

A double asterisk (**) denotes correlations significant at the 0.01 level and displayed in bold. A single asterisk (*) denotes correlations significant at the 0.05 level.

Table S2. Cross-test correlations obtained from bigels (BGs) formulated with gellan gum (GG) (n = 9) between rheological and mechanical properties and parameters derived from SAOS, steady-state, and penetration measurements. Variables from the same test are indicated in the same color.

	σ_{max}	γ_{max}	tan δ_{max}	a	b	E	G'	G''	η^*	tan δ	A	z	η_{10}	$\eta_{0.1}$	n	K	Recovery	F_{10}	F_B	S_B	W_{10}
σ_{max}	**1**	**-0.873****	-0.422	**0.979****	**0.967****	-0.780*	**0.996****	**0.994****	**0.996****	-0.694*	**0.998****	0.770*	**-0.803****	-0.336	**-0.944****	-0.668*	-0.522	**0.987****	**0.983****	**0.972****	**0.991****
γ_{max}		**1**	0.168	**-0.945****	-0.784*	**0.986****	**-0.899****	**-0.921****	**-0.901****	0.501	**-0.891****	-0.373	**0.989****	0.749*	0.738*	**0.944****	**0.864****	-0.792*	-0.784*	-0.748*	**-0.815****
tan δ_{max}			**1**	-0.290	-0.330	0.065	-0.422	-0.359	-0.416	**0.800****	-0.426	-0.618	0.099	-0.249	0.540	-0.048	-0.089	-0.454	-0.465	-0.572	-0.448
a				**1**	**0.926****	**-0.882****	**0.986****	**0.994****	**0.987****	-0.615	**0.984****	0.636	**-0.895****	-0.505	**-0.879****	-0.794*	-0.672*	**0.939****	**0.931****	**0.910****	**0.951****
b					**1**	-0.673*	**0.950****	**0.950****	**0.950****	-0.625	**0.955****	**0.828****	-0.710*	-0.199	**-0.940****	-0.553	-0.379	**0.981****	**0.981****	**0.945****	**0.971****
E						**1**	**-0.814****	**-0.844****	**-0.817****	0.395	**-0.804****	-0.213	**0.994****	**0.848****	0.621	**0.985****	**0.933****	-0.681*	-0.671*	-0.630	-0.712*
G'							**1**	**0.996****	**1.000****	-0.715*	**1.000****	0.735*	**-0.835****	-0.388	**-0.928****	-0.709*	-0.574	**0.971****	**0.966****	**0.959****	**0.977****
G''								**1**	**0.997****	-0.656	**0.995****	0.696*	**-0.863****	-0.437	**-0.917****	-0.747*	-0.609	**0.966****	**0.961****	**0.942****	**0.974****
η^*									**1**	-0.710*	**1.000****	0.732*	**-0.838****	-0.393	**-0.927****	-0.713*	-0.578	**0.971****	**0.966****	**0.958****	**0.977****
tan δ										**1**	-0.715*	-0.754*	0.426	0.015	0.685*	0.268	0.217	-0.691*	-0.691*	-0.774*	-0.689*
A											**1**	0.747*	**-0.825****	-0.371	**-0.933****	-0.696*	-0.559	**0.976****	**0.971****	**0.964****	**0.981****
z												**1**	-0.260	0.320	**-0.826****	-0.048	0.110	**0.848****	**0.852****	**0.878****	**0.823****
η_{10}													**1**	**0.820****	0.665	**0.970****	**0.916****	-0.710*	-0.702*	-0.658	-0.735*
$\eta_{0.1}$														**1**	0.141	**0.918****	**0.965****	-0.200	-0.188	-0.139	-0.244
n															**1**	0.502	0.331	**-0.952****	**-0.955****	**-0.957****	**-0.939****
K																**1**	**0.965****	-0.555	-0.544	-0.497	-0.589
Recovery																	**1**	-0.390	-0.375	-0.341	-0.430
F_{10}																		**1**	**0.999****	**0.985****	**0.998****
F_B																			**1**	**0.983****	**0.996****
S_B																				**1**	**0.983****
W_{10}																					**1**

A double asterisk (**) denotes correlations significant at the 0.01 level and displayed in bold. A single asterisk (*) denotes correlations significant at the 0.05 level.

Table S3. Correlations obtained between penetration measurements of bigels (BGs) and hydrogels (HGs) formulated with gelatin (GA) and beeswax-based oleogels (OGs) (n = 9). Variables from the same system are indicated in the same color.

	BG_F_{10}	BG_F_B	BG_S_B	BG_W_{10}	HG_F_{10}	HG_F_B	HG_S_B	HG_W_{10}	OG_F_{10}	OG_F_B	OG_S_B	OG_W_{10}
**BG_F_{10}**	**1**	**0.908****	**0.909****	**0.929****	**0.859****	**0.925****	**0.922****	**0.916****	0.364	0.117	0.664	0.762*
**BG_F_B**		**1**	**0.978****	**0.993****	**0.954****	**0.989****	**0.999****	**0.996****	0.450	0.174	**0.824****	0.774*
**BG_S_B**			**1**	**0.979****	**0.905****	**0.976****	**0.975****	**0.966****	0.461	0.082	0.792*	0.772*
**BG_W_{10}**				**1**	**0.956****	**0.980****	**0.995****	**0.994****	0.394	0.208	**0.841****	0.737*
**HG_F_{10}**					**1**	**0.910****	**0.958****	**0.972****	0.238	0.442	**0.920****	0.659
**HG_F_B**						**1**	**0.989****	**0.981****	0.546	0.046	0.737*	**0.827****
**HG_S_B**							**1**	**0.998****	0.442	0.186	**0.822****	0.776*
**HG_W_{10}**								**1**	0.410	0.234	**0.844****	0.760*
**OG_F_{10}**									**1**	-0.583	-0.032	0.705*
**OG_F_B**										**1**	0.616	-0.145
**OG_S_B**											**1**	0.415
**OG_W_{10}**												**1**

A double asterisk (**) denotes correlations significant at the 0.01 level and displayed in bold. A single asterisk (*) denotes correlations significant at the 0.05 level.

Table S4. Correlations obtained between penetration measurements of bigels (BGs) and hydrogels (HGs) formulated with gelatin (GG) and beeswax-based oleogels (OGs) (*n* = 9). Variables from the same system are indicated in the same color.

	BG_F_{10}	BG_F_B	BG_S_B	BG_W_{10}	HG_F_{10}	HG_F_B	HG_S_B	HG_W_{10}	OG_F_{10}	OG_F_B	OG_S_B	OG_W_{10}
BG_F_{10}	**1**	**0.999****	**0.985****	**0.998****	**0.954****	**0.987****	**0.939****	**0.963****	-0.630	0.013	-0.684*	**-0.865****
BG_F_B		**1**	**0.983****	**0.996****	**0.958****	**0.983****	**0.932****	**0.967****	-0.618	0.010	-0.686*	**-0.877****
BG_S_B			**1**	**0.983****	**0.939****	**0.965****	**0.910****	**0.913****	-0.532	-0.035	-0.719*	**-0.806****
BG_W_{10}				**1**	**0.947****	**0.990****	**0.950****	**0.957****	-0.659	0.054	-0.650	**-0.865****
HG_F_{10}					**1**	**0.899****	**0.811****	**0.900****	-0.499	-0.147	-0.774*	**-0.850****
HG_F_B						**1**	**0.980****	**0.966****	-0.716*	0.149	-0.576	**-0.859****
HG_S_B							**1**	**0.934****	**-0.811****	0.300	-0.421	**-0.840****
HG_W_{10}								**1**	-0.702*	0.095	-0.581	**-0.904****
OG_F_{10}									**1**	-0.583	-0.032	0.705*
OG_F_B										**1**	0.616	-0.145
OG_S_B											**1**	0.415
OG_W_{10}												**1**

A double asterisk (**) denotes correlations significant at the 0.01 level and displayed in bold. A single asterisk (*) denotes correlations significant at the 0.05 level.
